# HSA Adductomics in the Shanghai Women’s Health Study Links Lung Cancer in Never-Smokers with Air Pollution, Redox Biology, and One-Carbon Metabolism

**DOI:** 10.3390/antiox14030335

**Published:** 2025-03-13

**Authors:** Partow Imani, Hasmik Grigoryan, Sandrine Dudoit, Xiao-Ou Shu, Jason Wong, Luoping Zhang, Junfeng Zhang, Wei Hu, Qiuyin Cai, Yutang Gao, Batel Blechter, Mohammad Rahman, Wei Zheng, Nathaniel Rothman, Qing Lan, Stephen M. Rappaport

**Affiliations:** 1 School of Public Health, University of California, Berkeley, Berkeley, CA 94720, USA; pimani@berkeley.edu (P.I.); hasgrig@yahoo.com (H.G.); luoping@berkeley.edu (L.Z.); 2 Department of Statistics, University of California, Berkeley, Berkeley, CA 94720, USA; sandrine@stat.berkeley.edu; 3 Division of Epidemiology, Department of Medicine, Vanderbilt-Ingram Cancer Center, Vanderbilt University Medical Center, Nashville, TN 37232, USA; xiao-ou.shu@vanderbilt.edu (X.-O.S.); qiuyin.cai@vumc.org (Q.C.); wei.zheng@vumc.org (W.Z.); 4 Epidemiology and Community Health Branch, National Heart Lung and Blood Institute, National Institutes of Health, Department of Health and Human Services, Bethesda, MD 20892, USA; jason.wong@nih.gov; 5 Global Health Research Center, Duke Kunshan University, Kunshan 215316, China; junfeng.zhang@duke.edu; 6 Nicholas School of the Environment, Global Health Institute, Duke University, Durham, NC 27708, USA; 7 Division of Cancer Epidemiology and Genetics, National Cancer Institute, National Institutes of Health, Department of Health and Human Services, Bethesda, MD 20892, USA; wei.hu@nih.gov (W.H.); batel.blechter@nih.gov (B.B.); mohammad.rahman2@nih.gov (M.R.); rothmann@exchange.nih.gov (N.R.); qingl@mail.nih.gov (Q.L.); 8 Department of Epidemiology, Shanghai Cancer Institute, Renji Hospital, Shanghai Jiaotong University School of Medicine, Shanghai 200025, China; ytgao@vip.sina.com

**Keywords:** lung cancer, never-smokers, human serum albumin (HSA) adducts, cysteine 34 (Cys34), lysine 525 (Lys525), reactive oxygen species (ROS), polycyclic aromatic hydrocarbons (PAHs), fine particulate matter (PM_2.5_)

## Abstract

Nearly one fourth of lung cancers occur among never-smokers and are predominately lung adenocarcinomas (LUADs) that are distinct from smoking-related cancers. Causal links between LUADs in never-smokers have been attributed to reactive oxygen species (ROS) arising from airborne fine particulate matter (PM_2.5_) and polycyclic aromatic hydrocarbons (PAHs). These effects are pronounced among East Asian women who experience massive exposures to PM_2.5_ and PAHs and have the highest incidence of LUADs in the world. We employed untargeted adductomics to establish ROS adduct signatures in human serum albumin (HSA) from lung cancer cases and controls from never-smokers in the Shanghai Women’s Health Study. Forty-seven HSA adducts were quantified by mass spectrometry, nine of which were selected for association with lung cancer, including Cys34 sulfoxidation products and disulfides of cysteine and homocysteine and two modifications to Lys525. Associated adducts include constituents of redox biology and one-carbon metabolism (OCM), which are pathways associated with lung cancer. Differences in adduct abundance between cases and controls and correlations of adducts with urinary PAHs and dietary factors provide additional evidence linking air pollutants, OCM, and redox biology with lung cancer in never-smokers.

## 1. Introduction

Lung cancer is the leading cause of cancer mortality worldwide, accounting for 1.82 million deaths in 2022 (https://gco.iarc.fr/today/en/fact-sheets-cancers, accessed on 3 March 2024). Although most lung cancers are caused by tobacco smoking, nearly 25% occur among never-smokers and are predominately lung adenocarcinomas (LUADs) that are distinct from smoking-related cancers both histologically and epidemiologically [1]. In never-smokers, LUADs often contain oncogenic *EGFR* mutations, particularly among East Asian women (including those residing in the United States) that differ from those of never-smoking women in other geographic regions [1,2]. This implicates interactions across genetic factors, *EGFR* mutations and environmental effects as potential causes of lung cancer in never-smokers. Among never-smoking Chinese women, environmental risk factors for lung cancer include environmental tobacco smoke (ETS, secondhand smoke) [3], exposure to air pollutants [1,4] including cooking fumes [5], and dietary variables [6,7].

The International Agency for Research on Cancer (IARC) has declared air pollution to be carcinogenic based on evidence that combinations of pollutants and genetic factors cause lung cancers [8]. Across the plethora of air pollutants, fine particulate matter less than 2.5 μm in diameter (PM_2.5_) is regarded as a primary risk factor for LUADs in never-smokers [9]. Current exposures to PM_2.5_ in urban China are among the highest in the world with average PM_2.5_ concentrations in Shanghai exceeding 80 μg/m^3^, or roughly 20 times the World Health Organization (WHO) health guideline of 5 μg/m^3^ [1]. Following deposition in the deep lung, PM_2.5_ mobilizes inflammatory cells (alveolar macrophages and polymorphonuclear neutrophils) that generate reactive oxygen species (ROS) as a defense to kill invading microorganisms [10,11]. The same inflammatory response to nonviable PM_2.5_—including soot, fibers, transition metals, and cooking fumes—damages pulmonary epithelial cells and releases an array of cytokines including interleukin-1β (IL-1β) [10].

Based on an elegant set of experiments in which genetically engineered mice were induced with oncogenic human *EGFR^L858R^* and then exposed to relevant doses of PM_2.5_, Hill et al. demonstrated that macrophages are a primary source of IL-1β and that the signaling of this cytokine promotes *EGFR*-driven LUADs [9]. Then, using aggregate data from a set of epidemiological databases, including never-smokers, the authors reported an association between “... the frequency of *EGFR* mutant lung cancer incidence and increasing PM_2.5_ levels”. Based on these results and the literature, they concluded that the “... data suggest a mechanistic and causative link between air pollutants and lung cancer…”, with PM_2.5_ being an important tumor promoter. However, because tumors in mice exposed to PM_2.5_ did not contain increased numbers of *EGFR* mutant cells, they inferred that LUADs must be initiated by mutagenic co-exposures.

Prominent mutagenic exposures to air pollutants involve the ubiquitous mixture of polycyclic aromatic hydrocarbons (PAHs) adsorbed on soot particles arising from combustion processes as well as aerosols generated from the pyrolysis of cooking oils (both subsets of PM_2.5_) [12,13]. PAHs can cause cancer through pathways involving metabolism to mutagenic diol epoxides that form bulky DNA adducts and initiate tumors and to pairs of PAH catechols and quinones, metabolites that undergo redox cycling with the generation of tumor-promoting ROS [14,15]. Diesel engine exhaust (DEE) contains nanoparticles (diameters < 0.1 μm, also a subset of PM_2.5_) with adsorbed PAHs including mutagenic nitro-PAHs [16].

The hypothesis that exposure to PM_2.5_ and PAHs can cause lung cancer in never-smokers is supported by studies on inhabitants in Xuanwei, China, who were exposed to effluents from the indoor combustion of bituminous (“smoky”) coal, which contain massive concentrations of PM_2.5_ and PAHs [17]. Although women in Xuanwei are primarily nonsmokers, they nonetheless have a very high incidence of lung cancer [18] with LUAD tumors that harbor *EGFR* mutations [4,19,20]. Furthermore, Ho et al. conducted in vitro experiments exposing human LUAD cells, with and without *EGFR* mutations, to samples of PM_2.5_ from indoor coal combustion in Xuanwei and found a dose-related reduction in the viability of cells containing *EGFR-TKI* mutations [21]. Finally, Lan et al. reported that a single-nucleotide polymorphism in the *AKR1C3-Gln/Gln* genotype, which regulates the production of PAH catechols/quinones that generate ROS, was associated with increased incidence of LUADs in Xuanwei, particularly in never-smoking females [22].

In addition to the production of ROS by inflammatory cells, ROS are generated by nonphagocytic cells as signaling molecules for the oxidation and regulation of target proteins that promote critical cellular functions [23]. Under homeostasis, ROS are removed through reactions with cellular and extracellular antioxidants, of which low-molecular-weight thiols and protein thiols form the basis of redox biology [23]. When ROS production exceeds the body’s homeostatic defenses, a state of oxidative stress exists, which can be mitigated by antioxidants derived from cellular processes and diet. Indeed, there is evidence in both smokers and never-smokers for inverse associations between lung cancer and increased fruit and vegetable intake [24,25]. Increased dietary soy, which contains isoflavones with strong anti-inflammatory and antioxidant properties [26], was associated with reduced risk of lung cancer in never-smokers from the Shanghai Women’s Health Study (SWHS) [6]. An untargeted urinary metabolomics study of never-smokers in this population identified biological pathways associated with lung cancer, including oxidative stress, inflammation, and one-carbon metabolism (OCM) [27].

One-carbon metabolism represents a network of pathways involving folate, several B vitamins, homocysteine (hCys), and methionine and its derivative S-adenosylmethionine (SAM), a universal methyl donor for DNA and other molecules [28]. The methylation of DNA drives gene expression and the methionine–hCys cycle leads to the production of antioxidants including cysteine (Cys) and glutathione (GSH) [28]. Given the dynamic nature of OCM, various co-dependent molecules can become imbalanced, thereby altering redox homeostasis and the expression of genes that modulate the suppression and progression of cancers [19]. Pathways involving folate and methionine-hCys cycles were reported to reduce risks in lung cancer cases from the European Prospective Investigation into Cancer and Nutrition (EPIC) [29], and meta-analyses found that increased levels of folate and methionine in urine or blood were associated with reduced lung cancer risks [19,30].

The above evidence points to ROS as key intermediates in lung cancer etiology among never-smokers that involve the interplay between air pollutants, antioxidants, and redox biology. This motivates research to compare levels of ROS between never-smoking lung cancer cases and controls in prospective cohorts. Although ROS cannot readily be measured in vivo, blood levels can be inferred from their stable adducts in human serum albumin (HSA), which is the primary scavenger of ROS in the interstitial space. In particular, the Cys residue (Cys34) of the third largest tryptic (T3) peptide of HSA (^21^ALVLIAFAQYLQQC^34^PFEDHVK^41^, *m/z* 811.7593) is a nucleophilic locus that accounts for most of the antioxidant capacity [31]. Two Cys34 oxidation products (the sulfinic and sulfonic acids) are recognized biomarkers of oxidative stress [23,32], and Watanabe et al. investigated the clinical significance of the redox state of Cys34 to clinical findings involving inflammatory diseases of the liver and kidney and diabetes mellitus [33]. Furthermore, measuring adducts of the ε-amino group of the lysine residue Lys525 in another HSA peptide (^525^KQTALVELVK^534^, *m/z* 564.8529) expands coverage to different classes of adducts including Schiff bases and advanced glycation end products [34].

The aim of this study was to determine whether alterations to the Cys34/Lys525 adductome is associated with lung cancer risk in never-smokers from the SWHS, using plasma collected up to 14 years prior to diagnosis. By considering correlations among Cys34/Lys525 adduct features (hereafter simply called ‘features’) and covariates including biomarkers of PM_2.5_ and PAH exposure and dietary factors associated with lung cancer, we sought insight from this unique vantage point into the roles played by air pollution, oxidative stress, and OCM pathways in lung carcinogenesis.

## 2. Materials and Methods

### 2.1. Study Population

The SWHS is a prospective population-based cohort study on 74,942 women living in urban Shanghai, China, who were enrolled between the ages of 40 and 70 years in December 1996–May 2000 [35]. Within this larger prospective cohort study, we conducted a nested case/control study of 573 never-smoking women with available plasma samples including 278 incident lung cancer cases and 293 cancer-free controls. Controls were individually matched to cases on date of birth (±2 years) and date of sample collection (±3 months). Procedures for biospecimen collection and storage were previously described in detail. Participants completed a baseline survey, which collected information on demographics and anthropometric and lifestyle factors; dietary habits were gleaned from a food frequency questionnaire with a 7-day recall preceding blood collection.

### 2.2. Lung Cancer Ascertainment

The study participants were followed-up with using in-person surveys every 2–3 years and periodic linkage to the population-based Shanghai Cancer Registry, Shanghai Vital Statistics Registry, and Shanghai Resident Registry. In-home visits and a review of medical records by a panel of oncologists were used to verify all possible lung cancer diagnoses. Lung cancer was defined as code 162 under the *International Classification of Diseases*, *Ninth Revision* (*ICD-9*). All female lung cancer patients were captured upon diagnosis after recruitment through 31 December 2009. Lung cancer cases diagnosed before recruitment were excluded from the study. Data on lung cancer histological subtypes were extracted from medical records and classified according to code 8140/3 from the *International Classification of Diseases for Oncology*, *Second Edition* (*ICD-O-2*). Of the 278 incident lung cancer cases, we excluded from statistical analyses 72 cases that had not been histologically confirmed (HC), leaving 206 HC cases, of which 148 were either LUADs (133) or adenosquamous carcinomas (18) (combined hereafter as ‘LUADs’). The remaining HC cases included small-cell carcinomas (2), ‘other’ (3), ‘unclassified’ (22), and ‘missing ICD-O code’ (22).

### 2.3. Mass Spectrometry and Data Acquisition

Plasma samples were maintained at −80 °C for up to 14 years prior to analysis. Case/control pairs were analyzed randomly on the same day to reduce technical variation. Samples were processed and analyzed by nano-liquid chromatography–tandem mass spectrometry (nLC-HRMSMS) with duplicate injections, as detailed in Supplementary Method S1. After the tryptic digestion of HSA, an isotopically labeled T3 peptide modified with iodoacetamide (IAA-iT3) was added to normalize data for instrument performance. One microliter of the digest was then analyzed by nLC-HRMSMS to pinpoint Cys34 and Lys525 peptides. Precursor ions were extracted for monoisotopic peptide masses (MIMs) to assign accurate masses and elemental compositions. Peak areas of MIMs were normalized for HSA content by extracting the MIM of the doubly charged HSA peptide ^42^LVNEVTEFAK^51^ (‘housekeeping peptide’, *m/z* = 575.3111). Peaks were selected and integrated using average MIMs and retention times. Added masses relative to the Cys34 thiolate ion and the Lys525 peptide were estimated, and annotations were based on accurate mass, elemental composition, retention time, database searches, and a few reference standards.

Several urinary metabolites of exogenous PAHs and nitro-PAHs were measured by LC-MSMS, as described in Supplementary Method S2, for use as covariates in statistical analyses. Two composite sets of analytes were generated, namely ‘hydroxy-PAHs’ (sum of 2-, 3-, and 4-hydroxyphenanthrene, 1,9-dihydroxyphenanthrene, and 10-hydroxypyrene), which are metabolites of unmodified PAHs, and ‘amino-PAHs’ (sum of 9-aminophenanthrene, 3-aminobenzanthracene, 1- and 2-aminonaphthalene, 2-aminofluorene, and 1-aminopyrene), which are metabolites of nitro-PAHs, arising primarily from diesel emissions [16,36].

### 2.4. Statistical Analysis

Mass spectrometry data for 34 Cys34-containing features and 13 Lys525-containing features were available from 573 never-smoking subjects with fewer than 20 percent of missing observations. Duplicate injections were available for all but for two individuals who had single injections (*n* = 1144). Statistical analysis was conducted in the R environment (https://www.R-project.org/, version 4.2.2) and follows methods described previously [37]. Using a random effects model with the log feature abundance as the outcome, the subject-specific random effect and the random error term (from duplicate injections) were used to estimate the intraclass correlation coefficient (ICC) for each feature representing the proportion of variance that was not attributed to technical variation. As none of the ICC values were below an a priori cutoff of 0.2, no features were excluded. Duplicate injections were averaged while ignoring missing values, and features were sorted across subjects by the percentage of missing values. Three features with more than 30% missing values were removed after using Fisher’s exact test to check for differentially missing values by case/control status; this left 44 features for subsequent analysis. Any values that were still missing were imputed using the k-Nearest Neighbor algorithm with k equal to 4. The *scone* package (https://rdrr.io/bioc/scone/, accessed on 6 June 2023, R package version 1.22.0) was then used to evaluate various candidate normalization schemes based on several criteria measuring how well technical noise was removed while maintaining the signal of interest. The inputs to *scone* were the feature abundance matrix, a batch variable, the QC matrix (comprising the house-keeping peptide, internal standard, sample age, and run order), case–control status, and several candidate scaling methods [DESeq, Trimmed Mean of M-values, upper-quartile, and no scaling]. Based on *scone* results, the selected normalization procedure adjusted for all QC measures as well as batch and case/control status.

Following normalization, separate analyses were performed for comparing all HC cases to all controls and only LUAD cases (a subset of the HC cases) to all controls. A previously described ensemble method was applied to select the features that appeared to have the strongest association with the outcome for either HC cases or LUAD cases [37]. First, linear regression was used where each logged feature abundance was regressed on case/control status, batch, QC measures, and age, and the estimated regression coefficient corresponding to the case/control variable as well as the associated nominal *p*-value from the *t*-test were retained. Next, regularized logistic regression (LASSO) was used with the case/control status as the outcome and all normalized adducts and age as predictors. This was performed over 500 bootstrapped iterations, and the features were ranked based on the number of times their corresponding coefficients were non-zero. Features that ranked highly according to both nominal *p*-values from the linear regression and the bootstrapped LASSO were selected. Finally, the same inputs were given to Random Forests, and any additional features that appeared important, based on the mean decrease in Gini Index, were selected, thereby allowing for the detection of nonlinear relationships. Hierarchical clusterings of features using pairwise Spearman correlation were visualized with the R-package ‘*Superheat*’ (https://rlbarter.github.io/superheat/, accessed on 6 June 2023, R-package version 1.0.0). Heatmaps were similarly used to visualize feature correlations with various dietary factors and urinary PAH biomarkers as well as age, body mass index (BMI), and education level, and these variables were given to Random Forests to examine relative importance as predictors of case/control status.

Permutation tests were used to evaluate possible associations between relative feature abundance (case/matched control) and time from recruitment to diagnosis (ttd) to discriminate potentially causal effects from effects of disease progression (reverse causality) [37]. For each of 10,000 random permutations of the vector of the ttd (thereby breaking any association with relative case/control feature abundance), the estimated regression coefficient of case/control log-fold change (LFC) on the ttd was recorded. The *p*-value reported is the proportion of the 10,000 random permutations in which the absolute value of the estimated regression coefficient was greater than or equal to the absolute value estimated using the observed data. If a significant linear trend (either positive or negative) in LFC for a given feature was detected with increasing ttd, the feature was considered potentially reactive. The permutation test for each feature used its respective set of HC or LUAD cases and controls.

## 3. Results

Summary statistics are given in Table 1 comparing lung cancer cases and controls for covariates previously associated with lung cancer risk. Evidence of a significant inverse association between lung cancer and BMI was observed (*p*-value < 0.05), consistent with meta-analyses [38]. Soy consumption, age, and education level were also significantly lower in cases than controls. Table 1 also summarizes the ttd (years from recruitment).

The 34 Cys34 and 13 Lys525 features are listed in Table 2 and Table 3, respectively. For each feature, the following variables are shown: retention time, observed MIM, theoretical MIM, the corresponding mass deviation (Δmass), added mass, elemental composition, annotation, and mean peak area. Supplementary Table S1 shows the values of the ICC (median = 0.55; range: 0.25–0.93) and coefficient of variation (CV; median = 0.37; range: 0.18–1.4) based on the random effects model of duplicate nLC-HRMSMS analyses of feature abundances. The impact of technical variation in feature selection was reduced by averaging duplicate observations for each subject in the analyses. Supplementary Tables S2 and S3 show FCs, where the nominal *p*-values for testing whether the case/control coefficient from the multivariable regression are zero using a *t*-test for HC and LUAD cases, respectively, and all controls. In total, 34 of these 44 features have been reported previously in our laboratory (footnoted in Table 2 and Table 3).

Supplementary Figures S1A–C and S2A–C show the results of feature selection for HC cases/controls and LUAD cases/controls, respectively, and Table 4 combines the results to list the nine selected features. These included seven Cys34 features, i.e., 822.42 (Cys34 sulfinic acid), 827.088 (S-methanethiol), 845.42 (S-Cys-H_2_O), 849.07 (S-S-sulfonic acid trisulfide), 856.10 (S-hCys), 858.75 (Na adduct of S-Cys), and 914.83 (unknown), and two Lys525 features, i.e., 571.84 (Lys oxidation product; -H_2_+O) and 587.31 (unknown). Most of the selected features were common to both HC and LUAD analyses, exceptions being 822.42 (only for HC) and 849.07, 858.75, and 827.088 (only for LUADs). Also, two of the five features from the overlapping list (587.31 and 849.07) had stronger signals in the LUAD analysis despite the smaller sample size (148 cases as opposed to 206).

**Figure 1:**
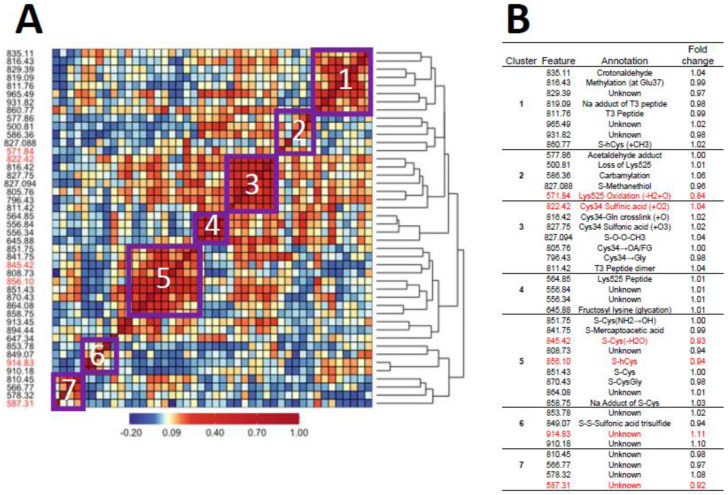
Heatmap of pairwise correlation matrix for all features in HC cases and controls (**A**) with annotations and case/control fold changes shown in (**B**). Features selected for association with lung cancer are highlighted in red.

**Figure 2:**
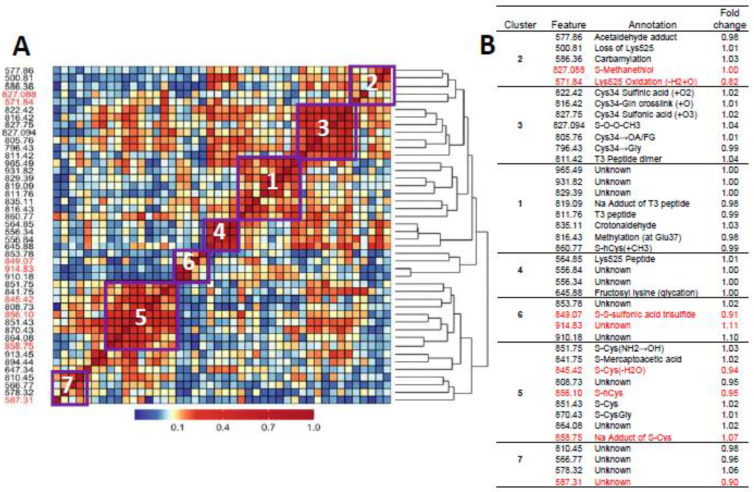
Heatmap of pairwise correlation matrix for all features in LUAD cases and controls (**A**) with annotations and case/control fold changes shown in (**B**). Features selected for association with lung cancer are highlighted in red.

Figure 1A and Figure 2A show heatmaps of pairwise Spearman correlation values for all features in the two analyses with selected features shown in red. Clusters labeled in the heatmaps are annotated in the embedded tables (Figure 1B and Figure 2B). The seven clusters in Figure 1 and Figure 2 between HC features and LUAD features, respectively, contain the same sets, although the relative locations differ in the heatmaps. This concordance of clusters again reflects the fact that LUADs represent 72 percent of HC cases.

**Figure 3:**
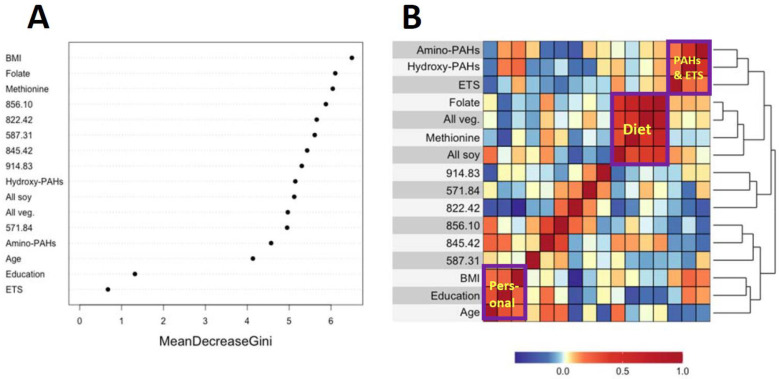
Variable importance of covariates and selected features as ranked by Random Forests in HC cases and controls (**A**) and heatmap showing the corresponding pairwise correlations (**B**).

**Figure 4:**
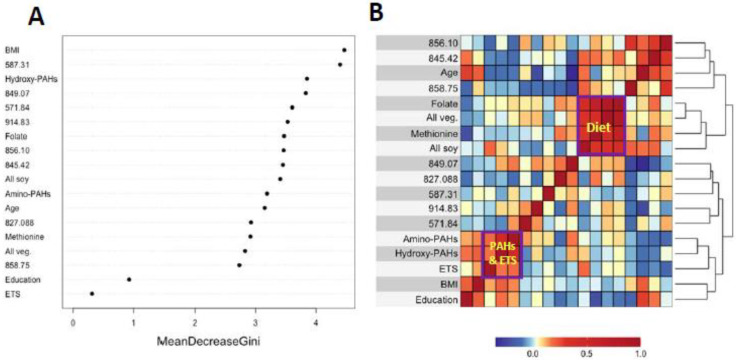
Variable importance of covariates and selected features as ranked by Random Forests in LUAD cases and controls (**A**) and heatmap showing the corresponding correlations (**B**).

Figure 3A and Figure 4A show variable importance plots created using the Random Forests of the selected features and covariates from HC and LUAD analyses side-by-side with heatmaps of pairwise Spearman correlation values (Figure 3B and Figure 4B). The BMI and methionine intake were the most important predictors of HC lung cancers followed closely by features 587.31, 914.83, and 822.42 (Figure 3A). The adjoining heatmap (Figure 3B) shows that all of the selected features except 914.83 were weakly to moderately correlated with urinary hydroxy-PAHs and/or amino-PAHs and that these correlations were negative in all cases. Feature 914.83 was negatively correlated with ‘all soy’ consumption, while 822.42 was negatively correlated with the entire dietary cluster (methionine, all soy, all vegetables and folate) as well as the ‘personal’ cluster representing education, age, and BMI. Turning now to the Random Forests plot for the features and covariates across LUADs in Figure 4A, BMI was the most predictive followed by 587.31, 849.07, and 571.84. As shown in Figure 4B, all selected features except 914.93 and 849.07 were weakly to moderately negatively correlated with one or both of the PAH classes. Also, the dietary cluster was positively correlated with 845.42, while 914.83 was negatively correlated with soy intake.

To determine whether potential associations of features with lung cancer may have resulted from reverse causality, we examined linear regressions between LFC of abundances of lung cancer case/control pairs and ttd. The results are presented in Supplementary Figures S3 and S4 as individual plots for the six selected features in HC cases/controls and eight selected features in LUAD cases/controls, respectively. There was no apparent linear trend for the ttd for any of the selected features in their respective subsets, indicating little evidence of reverse causality.

## 4. Discussion

### 4.1. Air Pollution and Lung Cancer in Never-Smokers

An increasing body of observational, mechanistic, and experimental evidence points to air pollution as a cause of lung cancer in never-smokers. A key element linking these lines of inquiry is the production of ROS by PM_2.5_ and PAHs, two ubiquitous classes of air pollutants that collectively initiate and promote lung cancers. A missing component of this amalgamation of findings is direct evidence that the production and removal of ROS in lung cancer cases is imbalanced in never-smokers. The Cys34/Lys525 adductome is an emerging biomarker of exogenous exposures and endogenous processes that provides a unique vantage point for linking adducts generated by ROS in vivo to lung cancer. Cys34 adducts of *N*-acetylcysteine, cysteinyl glycine (CysGly), and hCys were associated with lung cancer and smoking histories in the EPIC cohort [43] and in patients with chronic obstructive pulmonary disease (COPD, a risk factor for lung cancer) [42] but have not been investigated before in never-smoking lung cancer cases. In this study, we provide evidence that Cys34 adduct features do indeed link lung cancer in never-smokers with air pollution and altered redox biology and further implicate OCM as an important mechanistic pathway.

Our study focused on Chinese women because they have remarkably high lung-cancer incidence despite being predominately never-smokers. This has led to speculation that lung cancer risk across Chinese women reflects the confluence of high exposures to PM_2.5_ and PAHs—arising from the indoor/outdoor combustion of fossil fuels and household frying with cooking oils—and their interactions with genetic factors, somatic events, and tumor biology [1]. A key component to this conjecture is chronic inflammation resulting from the imbalance between the production of ROS by phagocytic cells (in response to PM_2.5_) [9,10] plus the redox cycling of PAH catechols/quinones [14,15] and the removal of ROS by cellular and extracellular antioxidants [46].

### 4.2. ROS Production and Removal

To investigate such imbalance between ROS production and removal in the SWHS, we monitored modifications to the Cys34/Lys525 adductome in 573 never-smoking women, including 278 incident lung cancer cases. Nine features were selected for association with lung cancer (Table 4) using a set of regression and classification methods, as applied previously with incident cases of non-Hodgkin’s lymphoma (NHL) and matched controls from the EPIC cohort [37]. Interestingly, the abundances of seven of these nine features were lower in cases than controls (0.823 ≤ FC ≤ 0.946), suggesting that enhanced ROS homeostasis in control subjects was protective for lung cancer.

Since seven of the nine selected features represent adducts to Cys34, we briefly differentiate between Cys34 sulfoxidation products and Cys34 disulfides with circulating thiols. When mercaptalbumin (HSA-Cys34-SH) encounters ROS, the sulfhydryl group is oxidized to its unstable sulfenic acid (Cys34-SOH), which can be further oxidized to sulfinic acid (Cys34-SO_2_H) and sulfonic acid (Cys34-SO_3_H). These sequential oxidations are largely irreversible and represent biomarkers of protein damage arising from oxidative stress. Indeed, the loss of the antioxidant capacity of mercaptalbumin due to the oxidation of Cys34 is a harbinger of chronic diseases of the liver and kidney as well as diabetes mellitus [33,47,48]. Thus, it is notable that the sulfinic acid feature (822.42; FC = 1.04) was selected for association with HC lung cancers and was among the most abundant features measured (Table 2). In cross-sectional investigations into Cys34 adductomics among residents of central London with high exposures to DEE, 822.42 was associated with COPD (FC = 0.85, *p* = 0.038), a risk factor for lung cancer, and ischemic heart disease (FC = 0.78, *p* = 0.027) [42]. The apparent reversal in FC values for 822.42 from negative in the COPD study to positive in our lung cancer investigation probably reflects differences in disease endpoints as well as the subjects’ geographic locations, age, ethnicity, smoking history, and diet. The other Cys34 sulfoxidation product selected for association with lung cancers in this study, namely, 849.07 (S-sulfonic acid trisulfide, FC = 0.91 for LUADs and 0.935 for HC cases) was observed in some of our previous investigations (footnoted in Table 2) but without notable associations. Although the origin of 849.07 is obscure, Kim et al. reported similar structures arising from reactions between a protein sulfinic acid and Cys with subsequent oxidations and degradation reactions [23].

In contrast to irreversible oxidations to its sulfinic and sulfonic acids, the sulfenic acid (Cys34-SOH) can react reversibly with circulating thiols (R-SH) to form disulfides (Cys34-S-S-R). Because Cys and hCys are among the most abundant low-molecular-weight thiols in the serum, it is not surprising that their Cys34 disulfides would be prominent features in our samples (Table 2), and three of them were selected for association with lung cancer (Table 4). Furthermore, the lower abundance of 845.42 [S-Cys(-H_2_O), FC = 0.93] and 856.10 (S-hCys, FC = 0.94) in never-smoking lung cancer cases from our study reinforces similar findings in both smokers and nonsmokers from the EPIC investigation, i.e., for S-Cys(-H_2_O) (FC = 0.71, *p* = 0.15) and S-hCys (FC = 0.74, *p* = 0.09) [43]. Interestingly, we previously used our classification methods to select S-Cys(-H_2_O) and S-hCys for association with exposure to DEE (FC = 0.88 and 0.91, respectively) [45] and also found evidence in never-smoking women in Xuanwei for associations between S-Cys(-H_2_O) and exposure to emissions from smoky coal (FC = 0.37, *p* = 0.07) and the corresponding air concentration of the carcinogenic PAH, benzo[a]pyrene (FC = 1.46, *p* = 0.022) [40]. These findings further suggest that exposure to PM_2.5_ and PAHs is a potential risk factor for lung cancer in never-smokers.

### 4.3. The Role of OCM in Lung Cancer

Since hCys is a key OCM intermediate involved in the production of methionine, and Cys and GSH together form much of the basis for redox homeostasis [28], the dysregulation of hCys metabolism may be a risk factor for lung cancer. Indeed, hCys is an important component of desulfuration and transsulfuration pathways that involve cystathionine β-synthase (CBS), an enzyme that catalyzes reactions producing Cys, methionine, GSH, and hydrogen sulfide (H_2_S) [49]. Acting through the modulation of hCys and H_2_S metabolism, CBS contributes to biological processes controlling redox homeostasis, DNA methylation and protein modification that, when perturbed, can lead to pathological consequences [49]. As noted previously, Seow et al. linked lung cancer among never-smokers from the SWHS to pathways involving methionine and Cys metabolism [27], and Baltar et al. reported protective effects of pathways involving methionine-hCys on lung cancer in EPIC [29]. Furthermore, the lower abundance of two of the three selected Cys and hCys features 845.42 [S-Cys(-H_2_O), FC = 0.93] and 856.10 (S-hCys, FC = 0.94) in never-smoking cases from our study as well as smokers and nonsmokers from the previous EPIC investigation [43] is consistent with the hypothesis that depletion of serum thiols is a risk factor for lung cancer. Interestingly, the Cys34 S-sulfonic acid trisulfide (849.07) does not cluster with the other Cys34 sulfoxidation products (cluster 3, Figure 1 and Figure 2) but is negatively correlated with the Cys and hCys disulfides in cluster 5, suggesting possible influence in depleting serum thiols and the dysregulation of hCys metabolism.

### 4.4. A Connection to Microbial Translocation?

The other annotated Cys34 feature selected for association with HC lung cancers was 827.088 (FC = 1.00), which is the Cys34 disulfide of methane thiol that is produced by enteric microbiota and was previously selected for associations with CRC (FC = 1.20) [44] and NHL (FC = 1.39, males only) [37] in EPIC. Given the elevation of this biomarker in NHL cases, it was hypothesized that the translocation of enteric bacteria from the gut resulted in the systemic release of ROS that can promote cancers throughout the body [37] (perhaps including the lung). However, this result from the current study should be regarded cautiously because the selection of 827.088 was based solely on Random Forests in the LUAD cases/controls and was not elevated in cases (Supplementary Figure S1C).

### 4.5. Influence of Covariates

Analyses of covariates by Random Forests, summarized in Figure 3A and Figure 4A, indicate that BMI was ranked highest in lung cancer cases and controls. This reflects the significantly lower values of BMI in cases here (Table 1) and in the literature [38]. Dietary methionine was also highly ranked in HC cases and controls (Figure 3A), which is consistent with elevated plasma methionine in lung cancer cases across observational studies [19] and with dysregulated methionine and Cys metabolism in never-smoking lung cancer cases in the SWHS [27]. Also, the high rank of the Cys34 sulfinic acid (822.42) in HC cases (Figure 3A) points to oxidative stress as a potentially causal factor and is reinforced in LUAD cases by the high ranks of the Cys34 S-sulfonic acid trisulfide (849.07) and hydroxy-PAHs (Figure 4A).

The heatmaps in Figure 3B and Figure 4B show correlations of selected features with covariates for HC and LUAD cases, respectively. All of the dietary variables (folate, vegetables, methionine, and soy) are positively correlated with each other, as are the two sets of PAH biomarkers and ETS (which contains PM_2.5_ and adsorbed PAHs). Several selected features were negatively correlated with the PAH/ETS cluster, namely the Cys34 disulfides of Cys (845.42), hCys (856.10), and Cys34 sulfinic acid (822.42) with HC cases (Figure 3B), suggesting that ROS generated by inhaled PM_2.5_ and PAH metabolism perturbed redox homeostasis. Again, of particular interest is 856.10 (hCys, FC = 0.94), which is negatively correlated with ETS and the amino-PAHs (metabolites of nitro-PAHs) that are biomarkers of DEE [16] but not with the hydroxy-PAHs derived from other combustion processes (Figure 3B and Figure 4B). Since we had previously found levels of 856.10 to be reduced in workers and urban residents exposed to DEE [42,45], this suggests that DEE may have a particular connection with lung cancer in urban never-smokers, possibly due to inflammation arising from nanoparticles and nitro-PAHs that are produced primarily by diesel engines [16]. The DEE connection with lung cancer may be explained by the strong mutagenicity of nitro-PAHs, as observed in our previous work [50]. Of the dietary variables, the most noteworthy finding is the strong negative correlation between the Cys34 sulfinic acid (822.42) and consumption of soy and methionine in HC cases (Figure 3B), which links dietary antioxidants with reduced systemic oxidative stress. Dietary methionine may be especially important because it plays a role in the formation of GSH via OCM and is itself a scavenger of ROS [51]. The personal cluster (BMI, age, and education) in HC cases also shows strong negative correlations with the Cys34 sulfinic acid (822.42) (Figure 3B), suggesting inverse associations with systemic oxidative stress as subjects become older, heavier, and better educated.

### 4.6. Strengths and Weaknesses

This investigation has several strengths. First, by focusing on never-smokers in the SWHS, we were able to investigate lung cancer risks in a particularly vulnerable population without the confounding effects of smoking. Second, the potential for reverse causality was reduced because plasma samples were collected prior to diagnosis by up to 14 years. Third, the untargeted HSA adductomics assay provides an unbiased and unique vantage point with which to evaluate ROS disposition during the month preceding phlebotomy and to determine whether ROS may have contributed to lung cancer. Fourth, using a hypothesis-free design and a set of complementary regression and classification methods, we reduced biases in selecting features for associations with lung cancer, including both linear and nonlinear relationships. Fourth, expanding the adductomics pipeline to Lys525 extended the underlying chemistries of adducts and included two selected features (571.84, Lys oxidation, and 587.32, unknown).

We also recognize some weaknesses. Plasma specimens were stored at −80 °C for up to 14 years prior to analysis, leading to the possibility that adducts may have been modified during storage. Such potential production of artifacts during storage was minimized by matching cases and controls by year of enrollment. Since only four of the nine selected features were confirmed with synthetic standards (822.42, 827.088, 856.10, and 858.75), the annotations of the others were based on elemental composition and should be regarded as putative. Also, two seemingly important features (587.31 and 914.83) were unannotated, limiting our ability to address their implications. Given a residence time of one month for human HSA adducts, levels measured in blood collected at recruitment may not accurately reflect those produced in subsequent years.

## 5. Conclusions

In conclusion, this untargeted adductomics investigation selected nine HSA adduct features that provided a unique vantage point for considering associations between ROS and lung cancer up to 14 years prior to diagnosis in never-smoking females (206 cases and 293 controls). The annotations of seven of these features point to both direct and indirect links among PM_2.5_ and PAH exposures, redox biology, OCM, and dietary antioxidants, consistent with the literature. Several of these features have previously been selected with similar effects in adductomic investigations into lung cancer and COPD and with environmental exposures to PM_2.5_, PAHs, and DEE. Future work should seek the validation of these findings with other lung cancer cohorts involving never-smokers and should apply this untargeted adductomic approach more generally to discover causes of other inflammatory diseases including atherosclerosis.

## Figures and Tables

**Table 1:** Summary statistics for covariates included with histologically confirmed (HC) cases, LUAD (lung adenocarcinoma) cases and controls.

Variable		HC Cases (*n* = 206)	LUAD Cases (*n* = 148)	Controls (*n* = 293)		*p*-Value (HC) ^1^	*p*-Value (LUAD) ^1^
BMI (kg/m^2^)	Min	16.51	16.59	Min	16.45	0.001 *	0.005 *
	Mean	23.82	23.82	Mean	24.90		
	Median	23.82	23.90	Median	24.44		
	Max	35.42	35.42	Max	36.18		
	Not available	0	0	Not available	1		
Amino-PAHs ^2^ (pg/mL)	Min	555.8	591.6	Min	288.1	0.226	0.449
	Mean	4783	4949	Mean	3916		
	Median	3544	3508	Median	2951		
	Max	57,121	57,121	Max	16,024		
	Not available	116	85	Not available	178		
Hydroxy-PAHs ^3^ (pg/mL)	Min	95.87	95.87	Min	105.5	0.820	0.840
	Mean	1040	1010	Mean	978.0		
	Median	872.0	854.4	Median	799.9		
	Max	3914	3914	Max	3578		
	Not available	126	95	Not available	189		
Education	College	30	21	College	34	0.024 *	0.003 *
	High School	60	50	High School	60		
	Middle School	57	39	Middle School	80		
	Elementary	59	38	Elementary	119		
Age (years)	Min	40	40	Min	40	0.002 *	0.0003 *
	Mean	56.2	55.5	Mean	58.5		
	Median	57	56.5	Median	62		
	Max	70	70	Max	70		
All Soy Consumption (g/d)	Min	6.29	14.42	Min	2.28	0.014 *	0.270
	Mean	211.3	236.8	Mean	249.6		
	Median	89.10	100.3	Median	151.3		
	Max	1099	1099	Max	1277		
All Vegetable Consumption (g/d)	Min	52.2	59.5	Min	23.8	0.487	0.075
	Mean	308.7	320.7	Mean	298.1		
	Median	265.0	294.7	Median	258.7		
	Max	1050	983.6	Max	878.6		
Methionine (g/d)	Min	0.414	0.414	Min	0.630	0.781	0.659
	Mean	1.501	1.522	Mean	1.505		
	Median	1.393	1.440	Median	1.454		
	Max	3.986	3.826	Max	3.167		
Folate (μg/d)	Min	45.3	45.3	Min	68.4	0.675	0.182
	Mean	263.6	269.8	Mean	264.3		
	Median	250.0	262.8	Median	244.0		
	Max	692.2	658.3	Max	1155		
ETS (Ever Exposed)	No	44	34	No	75	0.522	0.624
	Yes	141	133	Yes	204		
	Not available	21	15	Not available	14		
Time to Diagnosis (years)	Min	0.183	0.183				
	Mean	7.533	7.568				
	Median	8.355	8.475				
	Max	13.503	13.336				

* *p*-value < 0.05. ^1^ Nominal *p*-values for testing for differences in covariate distributions between cases (HC or LUAD) and controls were computed using a chi-squared test for categorical variables and a Wilcoxon rank-sum test for continuous variables. ^2^ Sum of 9-aminophenanthrene, 3-aminobenzanthracene, 1- and 2-aminonaphthalene, 2-aminofluorene, and 1-aminopyrene. ^3^ Sum of urinary 2-, 3-, and 4-hydroxyphenanthrene, 1,9-dihydroxyphenanthrene, and 10-hydroxypyrene.

**Table 2:** T3-peptide (mostly Cys34) features with sufficient data for statistical analysis. (Mean peak areas are based on normalized data after imputation for missing values.)

Feature	RT, min	MIM Observed (*m/z*, +3)	MIM Theoret. (*m/z*, +3)	ΔMass (ppm)	Added Mass (+H, Da)	Elemental Composition	Annotation	Mean Peak Area (×10^6^)
796.43 ^a,b,c,f,g,h^	27.717	796.4298	796.4301	−0.3767	−45.983	-CH_2_S	Cys34→Gly	6.16
805.76 ^a,b,c,f,h^	27.365	805.7614	805.7618	−0.4964	−17.989	-SH_2_, +O	Cys34→oxoalanine or fGly	0.182
808.73 ^a,b,c,d,e,f,g,h^	27.868	808.7281			−9.088		Not Cys34 adduct	42.9
810.45 ^a,d,f^	28.263	810.4521			−3.916		Not Cys34 adduct	0.168
811.42 ^a,b,c,d,e,f,g,h^	30.803	811.4242	811.4234	0.9859	−1.000		T3 dimer *	3.45
811.76 ^a,b,c,d,e,f,g,h^	28.682	811.7576	811.7594	−2.2174	0.000		Unmodified T3 *	9.25
816.42 ^a,b,c,d,e,f,g,h^	27.390	816.4188	816.4191	−0.3675	13.984	-H_2_, +O	Cys34-Gln crosslink *	1.39
816.43 ^a,b,c,d,e,f,g,h^	29.102	816.4306	816.4312	−0.7349	15.027	+CH_3_	Methylation (at Glu37)	3.94
819.09 ^b,e,g^	28.514	819.0863			22.994		Na adduct of T3	0.469
822.42 ^a,b,c,d,e,f,g,h^	27.207	822.4222	822.4226	−0.4864	33.002	+HO_2_	Cys34 sulfinic acid *	16.4
827.088 ^b,c,f,g,h^	29.566	827.0883	827.0886	−0.3627	47.000	+CH_3_S	S-Methanethiol *	40.9
827.094 ^c,d,f,g,h^	27.790	827.0942	827.0945	−0.3627	47.018	+CH_3_O_2_	S-(O)-O-CH_3_	1.23
827.75 ^a,b,c,d,e,f,g,h^	27.361	827.7537	827.7543	−0.7249	48.996	+HO_3_	Cys34 Sulfonic acid *	2.08
829.396 ^a,b,g,h^	28.575	829.3959			53.923		Not Cys34 adduct	0.321
835.11 ^a,c,e,g,f,h^	28.809	835.1062	835.1066	−0.4790	71.054	+C_4_H_7_O	Crotonaldehyde *	1.58
841.75 ^a,b,c,d,e,f,g,h^	28.411	841.7513	841.7519	−0.7128	90.989	+C_2_H_3_O_2_S	S-Mercaptoacetic acid	0.061
842.07	28.121	842.0734			91.955		Unknown	NA
845.42 ^a,b,c,d,e,f,g,h^	27.383	845.4238	845.4239	−0.1183	102.006	+C_3_H_4_NOS	S-Cys (-H_2_O)	0.487
849.07 ^a,b,c,h^	28.440	849.0684	849.0689	−0.5889	112.940	+HO_3_S_2_	S-Sulfonic acid trisulfide	0.880
851.43 ^a,b,c,d,e,f,g,h^	26.980	851.4266	851.4274	−0.9396	120.015	+C_3_H_6_NO_2_S	S-Cys *	67.2
851.76 ^a,b,c,d,e,f,g^	27.632	851.7554	851.7554	0.0000	121.000	+C_3_H_5_O_3_S	S-Cys (NH2→OH)	1.23
853.78 ^a,b,f^	27.503	853.7823			127.082		Unknown	9.20
856.10 ^a,b,c,d,e,f,g,h^	26.746	856.0988	856.0993	−0.5840	134.032	+C_4_H_8_NO_2_S	S-hCys *	200
858.75 ^a,b,c,d,e,g,h^	26.391	858.7546			141.999	+C_3_H_5_NO_2_SNa	Na adduct of S-Cys	1.52
860.77 ^b,d,e,f,g^	26.866	860.7703	860.7712	−1.0456	148.046	+C_5_H_10_NO_2_S	S-hCys (+CH_3_)	5.10
864.08 ^a,b,c,e,f,g^	26.305	864.0763			157.964		Not Cys34 adduct	0.760
870.43 ^a,b,c,d,e,f,g,h^	26.031	870.4342	870.4345	−0.3447	177.038	+C_5_H_9_N_2_O_3_S	S-CysGly *	0.751
894.44 ^a,b,c,d,e,f,g,h^	26.684	894.4414	894.4416	−0.2236	249.059	+C_8_H_13_N_2_O_5_S	S-γ-GluCys *	0.092
910.18 ^g,h^	32.954	910.1767			296.265	+C_18_H_34_NO_2_	Unknown	0.009
913.45 ^a,b,c,d,e,f,g,h^	26.554	913.4487	913.4487	0.0000	306.081	+C_10_H_16_N_3_O_6_S	S-Glutathione *	0.250
914.84 ^h^	32.931	914.8359			310.243		Unknown	0.552
931.82 ^b,c,d,f,g^	24.921	931.8204			361.196		Unknown	0.685
965.49 ^b,c,d,e,f,h^	25.207	965.4910			462.208		Unknown	2.24
970.16 ^d,f,h^	25.473	970.1631			476.224		Unknown	NA

Legend: MIM, monoisotopic mass; NA, not available; RT, retention time; fGly, formyl glycine. * Identity confirmed with a synthetic standard. ^a^ Reference [39]. ^b^ Reference [40]. ^c^ Reference [41]. ^d^ Reference [42]. ^e^ Reference [43]. ^f^ Reference [44]. ^g^ Reference [45]. ^h^ Reference [37].

**Table 3:** Lys525 peptide features with sufficient data for statistical analysis. (Mean peak areas are based on normalized data after imputation for missing values.)

Feature	RT, min	MIM Observed (*m/z*, +3)	MIM Theoretical (*m/z*, +3)	ΔMass (ppm)	Added Mass (+H, Da)	Elemental Composition (+H)	Annotation	Mean Peak Area (×10^8^)
500.81 ^a,b,c^	12.063	500.8045	500.8055	−1.9968	−128.095	-C_6_H_12_N_2_O	Loss of Lysine	156
556.34 ^a,b,c^	10.701	556.3396	556.3397	−0.1797	−17.024	-NH_3_	Loss of ammonia	0.037
556.84	10.995	556.8430	556.8436	−1.0775	−16.017	-NH_2_	Deamination	0.309
564.85 ^a,b,c^	10.822	564.8518	564.8529	−1.8474	0.000		Lys525 containing peptide	7.96
566.77 ^a,c^	10.684	566.7741			4.853		Unknown	0.014
571.84 ^c^	11.074	571.8423	571.8426	−0.5246	14.989	-H_2_, +O	Lys525 oxidation product	3.87
577.86 ^a^	12.454	577.8607	577.8608	−0.1731	27.026	+C_2_H_3_	Acetaldehyde	1.91
578.32	12.541	578.3164			27.937		Unknown	0.036
580.85 ^c^	11.110	580.8476	580.8479	−0.5165	32.999	+O_2_H	Lys525 oxidation product	NA
586.36 ^a,b,c^	13.265	586.3557	586.3559	−0.3411	44.016	+CH_2_NO	Carbamylation	9.59
587.31 ^c^	13.362	587.3109			45.926		Unknown	1.01
645.88 ^a,b,c^	10.904	645.8791	645.8794	−0.4645	163.063	+C_6_H_11_O_5_	Fructosyl lysine (glycation)/Hexose	18.4
647.34 ^c^	10.778	647.3378			165.980		Unknown	1.03

Legend: MIM, monoisotopic mass; NA, not available; RT, retention time. ^a^ Reference [34]. ^b^ Reference [45]. ^c^ Reference [37].

**Table 4:** Features selected by the ensemble pipeline for association with lung cancer. Selection criteria satisfied for each feature are shown in boldface. [Fold-change based on normalized and imputed data (FC); linear regression nominal *p*-value; bootstrap LASSO; Random Forests (RF)].

		Histologically Confirmed				LUAD			
Feature	Annotation	FC	*p*-Value	LASSO	RF	FC	*p*-Value	LASSO	RF
571.84	Lys525 oxidation (-H_2_+O)	0.842	**0.040**	**Yes**	**Yes**	0.823	**0.095**	**Yes**	**Yes**
587.31	Unknown	0.921	**0.081**	**Yes**	No	0.896	**0.040**	**Yes**	No
822.42	Cys34 sulfinic acid	1.042	**0.096**	**Yes**	**Yes**	1.018	0.504	No	No
827.088	S-Methanethiol	0.963	0.453	No	No	0.999	0.922	No	**Yes**
845.42	S-Cys(-H_2_O)	0.932	**0.014**	**Yes**	**Yes**	0.937	**0.047**	**Yes**	**Yes**
849.07	S-Sulfonic acid trisulfide	0.935	**0.079**	No	No	0.912	**0.028**	**Yes**	No
856.10	S-hCys	0.942	**0.044**	**Yes**	**Yes**	0.946	**0.087**	**Yes**	**Yes**
858.75	Na adduct of S-Cys	1.029	0.400	No	No	1.070	**0.087**	**Yes**	No
914.83	Unknown	1.114	**0.047**	**Yes**	**Yes**	1.113	**0.073**	**Yes**	**Yes**

## Data Availability

The datasets generated and/or analyzed during the current study are available from the corresponding author on reasonable request.

## Supplementary Materials

The following supporting information can be downloaded at: https://www.mdpi.com/article/10.3390/antiox14030335/s1. References [52,53] are cited in Supplementary Materials.

## Abbreviations

BMI, body mass index; CBS, cystathionine β-synthase; COPD, chronic obstructive pulmonary disease; Cys34, cysteine residue at position 34 of HSA; CysGly, cysteinyl glycine; DEE, diesel engine exhaust; *EGFR*, Epidermal Growth Factor Receptor; EPIC, European Prospective Investigation into Cancer and Nutrition; ETS, environmental tobacco smoke; FC, fold change; GSH, glutathione; HC, histologically confirmed lung cancer cases; hCys; homocysteine; HSA, human serum albumin; IARC, International Agency for Research on Cancer; IL-1β, interleukin-1β; ICC, intraclass correlation coefficient; LFC, log-fold change; LUAD, lung adenomas; LASSO, regularized logistic regression; Lys525, lysine residue at position 525 of HSA; MIM, monoisotopic mass; nLC-HRMSMS, nano-liquid chromatography–tandem mass spectrometry; OCM, one-carbon metabolism; ROS, reactive oxygen species; PAHs, polycyclic aromatic hydrocarbons; PM_2.5_, airborne fine particulate matter less than 2.5 μm in diameter; RF, Random Forests; SAM, S-adenosylmethionine; SWHS, Shanghai Women’s Health Study; T3 peptide; third largest tryptic peptide of HSA; ttd, time from subject recruitment to diagnosis; WHO, World Health Organization.
