# Supplementary material for: HSA Adductomics in the Shanghai Women’s Health Study Links Lung Cancer in Never-Smokers with Air Pollution, Redox Biology, and One-Carbon Metabolism
Source: Antioxidants (Basel). 2025 Mar 13;14(3):335. doi: 10.3390/antiox14030335 (PMC11939640; doi:10.3390/antiox14030335)
Supplement: Supplementary file 1 [file antioxidants-14-00335-s001.zip › antioxidants-3493959-supplementary.pdf]

Supplementary Materials

HSA Adductomics in the Shanghai Women's Health Study Links Lung Cancer in Never-Smokers with Air Pollution, Redox Biology and One-Carbon Metabolism

Authors: Partow Imani, Hasmik Grigoryan, Sandrine Dudoit, Xiaoou Shu, Jason Wong, Luoping Zhang, Junfeng (Jim) Zhang, Wei Hu, Qiuyin Cai, Yutang Gao, Batel Blechter, Mohammad Rahman, Wei Zheng, Nathaniel Rothman, Qing Lan and Stephen M. Rappaport

Supplementary Methods S1	Measurement of Cys34/Lys525 adducts using nano-liquid chromatography high-resolution tandem mass spectrometry (nLC-HRMSMS)	pp. 2-3
Supplementary Methods S2	LC-MS/MS analysis of urinary metabolites of hydroxy-polycyclic aromatic hydrocarbons (hydroxy-PAHs) and amino-PAHs.	p. 4
Supplementary Table S1	Intraclass correlation coefficients (ICC) and coefficients of variation (CV) from the random effects model for feature abundances from duplicate nLC-HRMSMS injections.	pp. 5-6
Supplementary Table S2	Case/control fold changes (FC) and nominal *p*-values after normalization and imputation for histologically confirmed (HC) lung cancer cases and controls.	pp. 7-8
Supplementary Table S3	Case/control fold changes (FC) and nominal *p*-values after normalization and imputation for lung adenoma (LUAD) cases and controls.	pp. 9-10
Supplementary Figure S1	Ensemble of regression and classification methods for selection of adduct features associated with case/control status for HC cases.	p. 11-13
Supplementary Figure S2	Ensemble of regression and classification methods for selection of adduct features associated with case/control status for LUAD cases.	p. 14-16
Supplementary Figure S3	Plots of ln(case/matched-control fold change) versus time to diagnosis (ttd) in years from recruitment for adduct features selected for HC lung cancer cases/controls.	p. 17
Supplementary Figure S4	Plots of ln(case/matched-control fold change) versus time to diagnosis (ttd) in years from recruitment for adduct features selected for LUAD cases/controls.	pp. 18-19

Supplementary Methods S1. Measurement of Cys34/Lys525 adducts using nLC-HRMSMS as summarized from [1-2].

Nano-liquid chromatography-high resolution mass spectrometry/mass spectrometry (nLC-HRMSMS) was performed in data-dependent mode with duplicate injections as described in [1-2] (References 39 and 34, respectively, in the text). Purified HSA (≥ 75 %) from 5 µL of plasma was digested with trypsin without prior reduction of disulfide bonds at 37°C using high-pressure cycling for 30 min (NEP2320, Pressure Biosciences Inc., South Easton, MA, USA). Prior to nLC-HRMSMS, 1 µL (20 pmol) of an internal standard, consisting of the isotopically labeled T3 peptide modified at Cys34 with iodoacetamide (IAA-iT3), was added to normalize data for instrument performance. One microliter of each digest was then injected into the nLC-HRMSMS, consisting of a Dionex Ultimate® 3000 nanoflow LC system equipped with a Dionex PepSwift monolithic column (100-µm i.d. x 25 cm) and connected via a Flex Ion nano-electrospray-ionization source with an Orbitrap Elite Hybrid HRMS (Thermo Scientific, Sunnyvale, CA, USA) that was operated in positive-ion mode with a spray voltage of 2.0 kV, a capillary temperature of 200°C and voltage of 35 V. Peptides were separated at a flow rate of 750 nL/min. Mobile phase A was water/0.1% formic acid (v/v) and mobile phase B was acetonitrile/0.1% formic acid (v/v). The gradient involved isocratic flow at 2% B for 5 min, a linear gradient from 2% to 45% B over 30 min, rapid increase to 98% B to wash the column (3 min), and reset to initial conditions over 5 min. The retention time range for Lys525 containing miss-cleaved peptides was set from 7 to 17 min compared to that of the Cys34 peptides (25 to 35 min). The precursor mass range for the search of triply-charged precursors of Cys34 adducts and doubly-charged precursors of Lys525 adducts was from 500 *m/z* to 1000 *m/z* which represents added masses from -129 to 370 Da and -185 to 565 Da for Lys525 and Cys34, respectively (negative added masses reflect losses due to elimination reactions, deletions, rearrangements and truncations). After sample injections, blanks and column washes were injected to maintain instrument performance as described in [1].

The mass spectra were acquired over the range m/z = 300−1200 using the Orbitrap mass analyzer (Thermo Scientific, Sunnyvale, CA, USA) in positive profile mode, with a resolution of 6×10^4 at *m/z* = 400. In data-dependent mode, up to 12 triply-charged (Cys34) and doubly-charged (Lys525) precursor ions exceeding 15,000 counts were selected from each MS1 scan and fragmented by collision-induced dissociation (CID) and analyzed in the Velos Pro linear ion trap in centroid mode. To avoid the occurrence of redundant MS2 measurements, real-time dynamic exclusion was enabled using the following parameters: repeat count 2, repeat duration 20 s, exclusion list size 500, exclusion duration 75 s, and exclusion precursor ion width ±10 ppm. Charge-state screening and monoisotopic-ion selection were enabled. The lock-mass option was enabled to provide a real-time internal mass calibration using a reference list of 8 identified background ions [3] (Reference 52 in the text). External mass calibration of the mass spectrometry and the LC diagnostics were performed weekly. MS1 and MS2 spectra were acquired and analyzed using Xcalibur software (version 3.0.63) and Chromeleon Xpress (v. 6.80) (Thermo Fisher Scientific, Sunnyvale, CA, USA). Adducts were located via MS2 spectra on the triply charged T3 peptide, that contains Cys34, (^{21}ALVLIAFAQYLQQC$^{\mathbf{34}}$PFEDHVK^{41}, *m/z* 811.7593) and the doubly charged miscleaved Lys525 peptide (^{525}KQTALVELVK^{534}, *m/z* 564.8529) that displayed the signature b^+- and/or y^{2+}-series ions [1]. The corresponding precursor ions were then extracted from the total ion chromatogram (TIC) to obtain a monoisotopic mass (MIM) for each adduct feature. To normalize peak areas for the amount of HSA in each sample, the MIM was also extracted for the HK peptide ^{42}LVNEVTEFAK^{51} (*m/z*=575.3111), which is adjacent to T3. The peak area ratio (PAR), representing the ratio of the abundance of the adduct peak to that of HK is a robust measure of the adduct concentration. Peak picking and integration were performed using the Xcalibur Processing Method (version 3.0, Thermo Fisher Scientific, Waltham, MA, USA) based on the average MIMs and retention times. Peak integration employed the Genesis algorithm after normalizing for instrument performance via iT3-IAA. Added masses relative to the Cys34 thiolate ion were estimated as $M_{adduct} = (m/z_{adduct} - m/z_{T3\text{-}peptide})*3 + 1.0078$, where m/z_{adduct} and $m/z_{T3\text{-}peptide}$ are the observed *m/z* values for the triply-charged MIMs of a given precursor ion for an adduct and the unmodified T3 peptide, respectively, and 1.0078 is the mass of a hydrogen atom.

The mass added to the ε-amino group of Lys525 was calculated in two ways to account for different mechanisms of adduct formation. The first calculation of added mass is consistent with formation of Michael-

addition products that can occur in HSA at Lys525. In this case, the MIM of the doubly charged precursor ion is subtracted from the corresponding MIM of the modified Lys525-containing miss-cleaved peptide and the difference is multiplied by two and the mass of one hydrogen atom is added. The second calculation applies to Schiff-base formation that can occur with the ε-amino group of Lys525 and involves loss of a water molecule. To contend with this mechanism, added masses for putative Lys525 adducts were calculated corresponding to addition of two hydrogens to Lys525. Notably, the added mass representing the reactive electrophile per se would show loss of the oxygen atom. All data processing utilized in-house software written in R.

References

1. Grigoryan H, Edmands W, Lu SS, Yano Y, Regazzoni L, Iavarone AT, et al. Adductomics Pipeline for Untargeted Analysis of Modifications to Cys34 of Human Serum Albumin. Anal Chem 2016;88(21):10504-12 doi 10.1021/acs.analchem.6b02553.
2. Grigoryan H, Imani P, Dudoit S, Rappaport SM. Extending the HSA-Cys34-Adductomics Pipeline to Modifications at Lys525. Chem Res Toxicol 2021;34(12):2549-57 doi 10.1021/acs.chemrestox.1c00311.
3. Keller BO, Sui J, Young AB, Whittal RM. Interferences and contaminants encountered in modern mass spectrometry. Anal Chim Acta 2008;627(1):71-81 doi 10.1016/j.aca.2008.04.043.

Supplementary Methods S2. LC-MS/MS analysis of urinary metabolites of exogenous and amino-PAHs as summarized from [4,5].

Several urinary metabolites of exogenous PAH and nitro-PAH were measured by LC-MSMS as described by Yang et al.[4] and Xu et al.[5] (References 36 and 43, respectively in the text) with modifications. Two composite sets of urinary PAH metabolites were generated from these measurements, namely hydroxylated-PAH metabolites ('Hydroxy-PAHs': sum of 2-, 3-, and 4-hydroxyphenanthrene, 1,9-dihydroxyphenanthrene, and 10-hydroxypyrene) that are products of metabolism of unmodified PAHs, and aminated PAHs ('Amino-PAHs': sum of 9-aminophenanthrene, 3-aminobenzanthracene, 1-, and 2-aminonaphthalene, 2-aminofluorene, and 1-aminopyrene) that are metabolites of nitro-PAHs.

For analysis of these PAH features, two mL of urine were incubated with 20 µL of β-glucuronidase from Helix pomatia Type H-2 (Sigma-Aldrich, St. Louis, MO, USA) in 2 mL of 0.1 M Na Acetate buffer (pH 5.0) at 37°C overnight. A sample enrichment and purification cartridge, packed with C18 was used for sample extraction and was eluted with 6 mL of methanol. The eluent was concentrated by evaporation and 20 µL was injected into the LC-MS/MS system consisting of a Nova-Pak C18 column (Waters 3.9 x 150 mm) (Waters corporation, Milford, Massachusetts, USA) and a Thermo TSQ Quantum Access Max triple quadrupole mass spectrometer with an electrospray ionization (Thermo Scientific, Sunnyvale, CA, USA) interface operated in negative ion mode with a spray voltage of 4 kV. Analytes were separated with a methanol/water mobile phase at a flow rate of 0.2 mL/min using stepwise elution starting with 55% methanol for 2 min., followed by 80% methanol for 25 min., then returning to 55% methanol for 10 min. The capillary temperature was optimized at 300°C, the sheath-gas pressure was 10 arbitrary units, and the auxiliary gas pressure was 5 arbitrary units. The selected reaction monitoring (SRM) transitions m/z 193.12/165.08 for hydroxyphenanthrenes and 199.20/171.20 for $^{13}C_6$ 3-hydroxyphenanthrene (internal standard) were used for detection.

References

4. Yang Z, Lin Y, Wang S, Liu X, Cullinan P, Chung KF, et al. Urinary Amino-Polycyclic Aromatic Hydrocarbons in Urban Residents: Finding a Biomarker for Residential Exposure to Diesel Traffic. Environmental science & technology 2021;55(15):10569-77 doi 10.1021/acs.est.1c01549.
5. Xu X, Zhang J, Zhang L, Liu W, Weisel CP. Selective detection of monohydroxy metabolites of polycyclic aromatic hydrocarbons in urine using liquid chromatography/triple quadrupole tandem mass spectrometry. Rapid Commun Mass Spectrom 2004;18(19):2299-308 doi 10.1002/rcm.1625.

Supplementary Table S1. Intraclass correlation coefficients (ICC) and coefficients of variation (CV) from the random effects model for feature abundances from duplicate nLC-HRMSMS injections.

**Feature**	**ICC**	**CV**
500.81	0.497	0.285
556.34	0.904	0.291
556.84	0.742	0.300
564.85	0.614	0.254
566.77	0.387	0.556
571.84	0.655	1.067
577.86	0.348	0.483
578.32	0.923	0.267
580.85	0.492	1.429
586.36	0.352	0.638
587.31	0.875	0.270
645.88	0.756	0.234
647.34	0.577	0.444
796.43	0.793	0.197
805.76	0.675	0.238
808.73	0.889	0.308
810.45	0.501	0.354
811.76	0.715	0.254
811.42	0.915	0.316
816.42	0.498	0.372
816.43	0.518	0.388
819.09	0.492	0.444
822.42	0.619	0.242
827.09	0.448	0.512
827.10	0.425	0.370
827.75	0.579	0.312
829.39	0.430	0.387
835.11	0.929	0.322
841.75	0.465	0.403
842.07	0.384	0.432
845.42	0.611	0.348
849.07	0.445	0.447
851.43	0.771	0.185
851.75	0.398	0.437
853.78	0.529	0.264
856.10	0.679	0.255
858.75	0.555	0.397
860.77	0.548	0.334
864.08	0.501	0.481
870.43	0.704	0.280
894.44	0.260	0.519

910.18	0.408	0.795
913.45	0.427	0.490
914.83	0.335	0.732
931.82	0.629	0.388
965.49	0.721	0.446
970.16	0.370	0.421
**Median**	**0.552**	**0.380**

Supplementary Table S2. Case/control fold changes (FC) and nominal *p*-values after normalization and imputation for HC lung cancer cases and controls.

Feature	FC	*p*-value
500.81	1.015	0.392
556.34	1.012	0.616
556.84	1.010	0.666
564.85	1.014	0.459
566.77	0.972	0.512
571.84	0.842	0.040
577.86	1.003	0.918
578.32	1.085	0.299
586.36	1.059	0.218
587.31	0.921	0.081
645.88	1.009	0.760
647.34	1.058	0.168
796.43	0.985	0.661
805.76	0.995	0.868
808.73	0.938	0.185
810.45	0.978	0.465
811.76	0.993	0.792
811.42	1.038	0.394
816.42	1.015	0.617
816.43	0.993	0.855
819.09	0.975	0.488
822.42	1.042	0.096
827.09	0.963	0.453
827.10	1.040	0.187
827.75	1.017	0.607
829.39	0.973	0.417
835.11	1.037	0.236
841.75	0.993	0.857
845.42	0.932	0.014
849.07	0.935	0.079
851.43	1.003	0.905
851.75	1.005	0.899
853.78	1.022	0.279
856.10	0.942	0.044
858.75	1.029	0.400
860.77	1.017	0.586
864.08	1.008	0.828
870.43	0.984	0.616
894.44	0.988	0.749
910.18	1.104	0.097
913.45	1.007	0.871

914.83	1.114	0.047
931.82	0.976	0.538
965.49	1.021	0.529

Supplementary Table S3. Case/control fold changes (FC) and nominal *p*-values after normalization and imputation for LUAD cases and controls.

Feature	FC	p-value
500.81	1.014	0.471
556.34	1.006	0.804
556.84	1.004	0.864
564.85	1.013	0.508
566.77	0.956	0.344
571.84	0.823	0.040
577.86	0.985	0.671
578.32	1.060	0.520
586.36	1.033	0.539
587.31	0.896	0.040
645.88	1.005	0.865
647.34	1.050	0.297
796.43	0.989	0.764
805.76	1.014	0.671
808.73	0.950	0.352
810.45	0.978	0.503
811.76	0.989	0.715
811.42	1.044	0.374
816.42	1.012	0.718
816.43	0.979	0.603
819.09	0.981	0.629
822.42	1.018	0.504
827.09	0.999	0.992
827.10	1.035	0.291
827.75	1.024	0.522
829.39	1.003	0.939
835.11	1.027	0.467
841.75	1.024	0.583
845.42	0.937	0.047
849.07	0.912	0.028
851.43	1.022	0.394
851.75	1.030	0.444
853.78	1.023	0.306
856.10	0.946	0.087
858.75	1.070	0.087
860.77	0.988	0.685
864.08	1.019	0.650
870.43	1.014	0.691
894.44	0.995	0.900
910.18	1.106	0.127
913.45	1.011	0.797

914.83	1.113	0.073
931.82	1.004	0.921
965.49	1.009	0.808

A

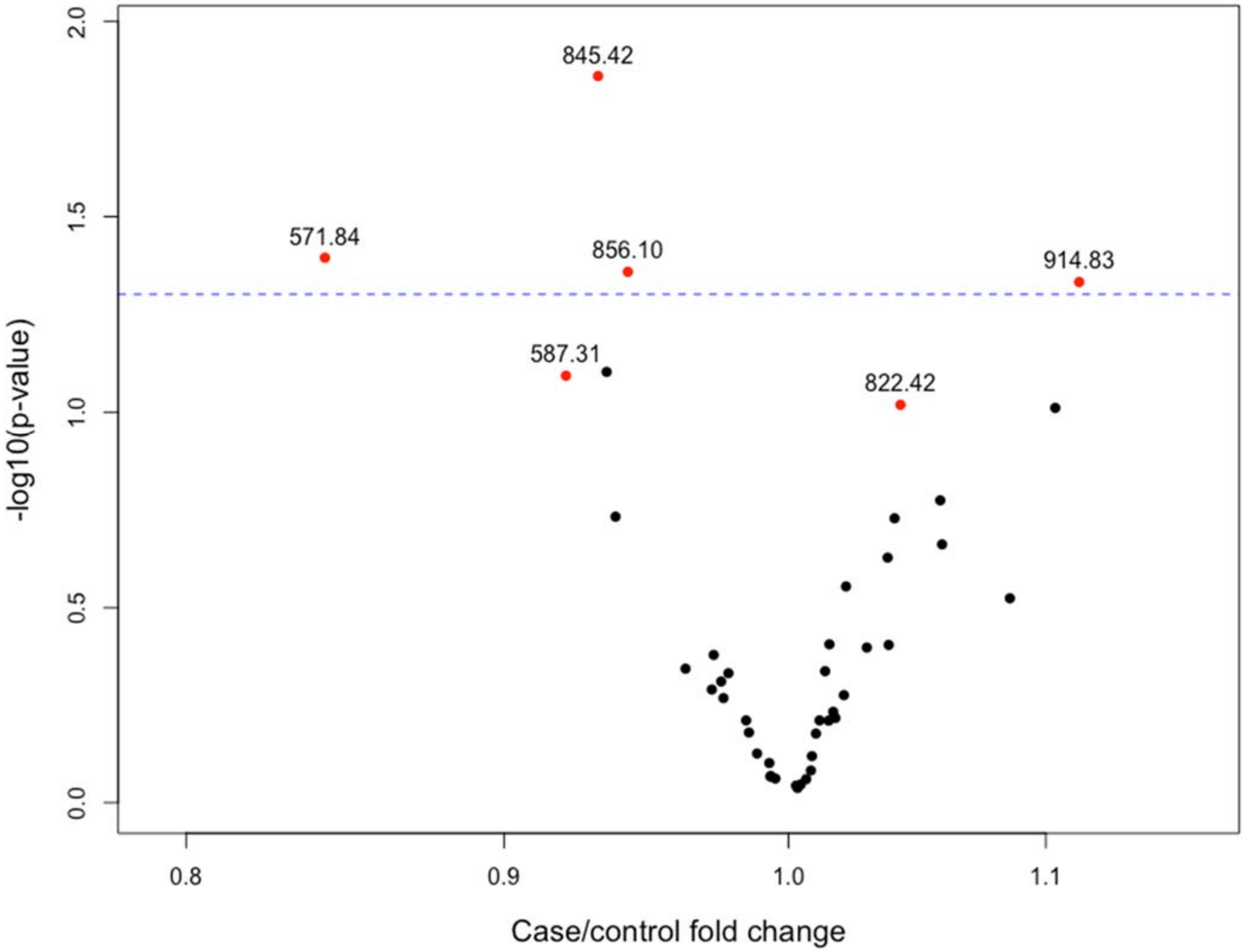

845.42
571.84
856.10
914.83
587.31
822.42
-log10(p-value)
0.0
0.5
1.0
1.5
2.0
0.8
0.9
1.0
1.1
Case/control fold change

B

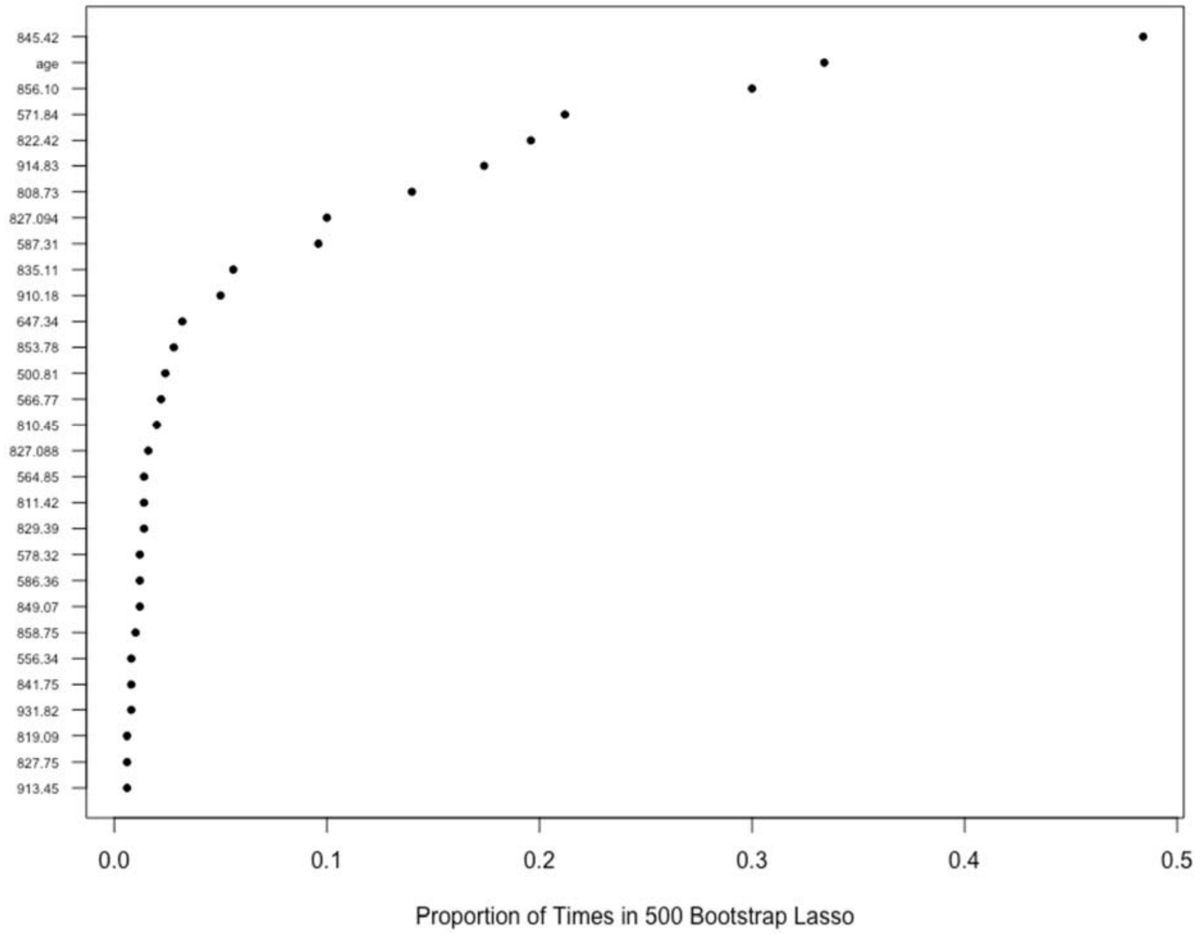
845.42
age
856.10
571.84
822.42
914.83
808.73
827.094
587.31
835.11
910.18
647.34
853.78
500.81
566.77
810.45
827.088
564.85
811.42
829.39
578.32
586.36
849.07
858.75
556.34
841.75
931.82
819.09
827.75
913.45
0.0
0.1
0.2
0.3
0.4
0.5
Proportion of Times in 500 Bootstrap Lasso

C

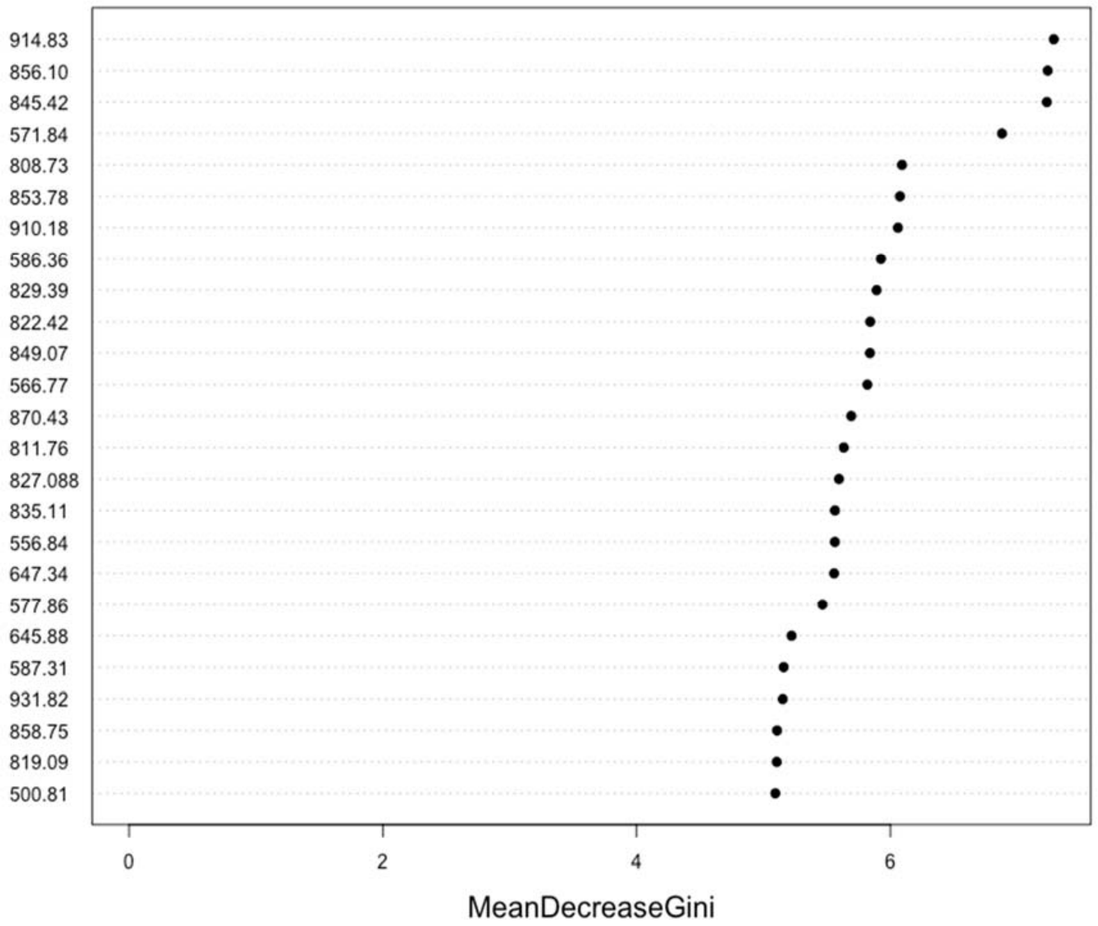

Supplementary Figure S1. Ensemble of classification models for selection of adduct features associated with lung-cancer incidence in histologically-confirmed cases and controls. A) Volcano plot of nominal *p*-values for case status in multivariate linear regressions of each adduct with selected feature shown in red (dashed line indicates *p*=0.05); B) proportion of times that a given feature was selected by a regularized logistic regression (LASSO) of NHL case status; C) ranked variable importance measures from a Random Forest classification of case-control status.

A

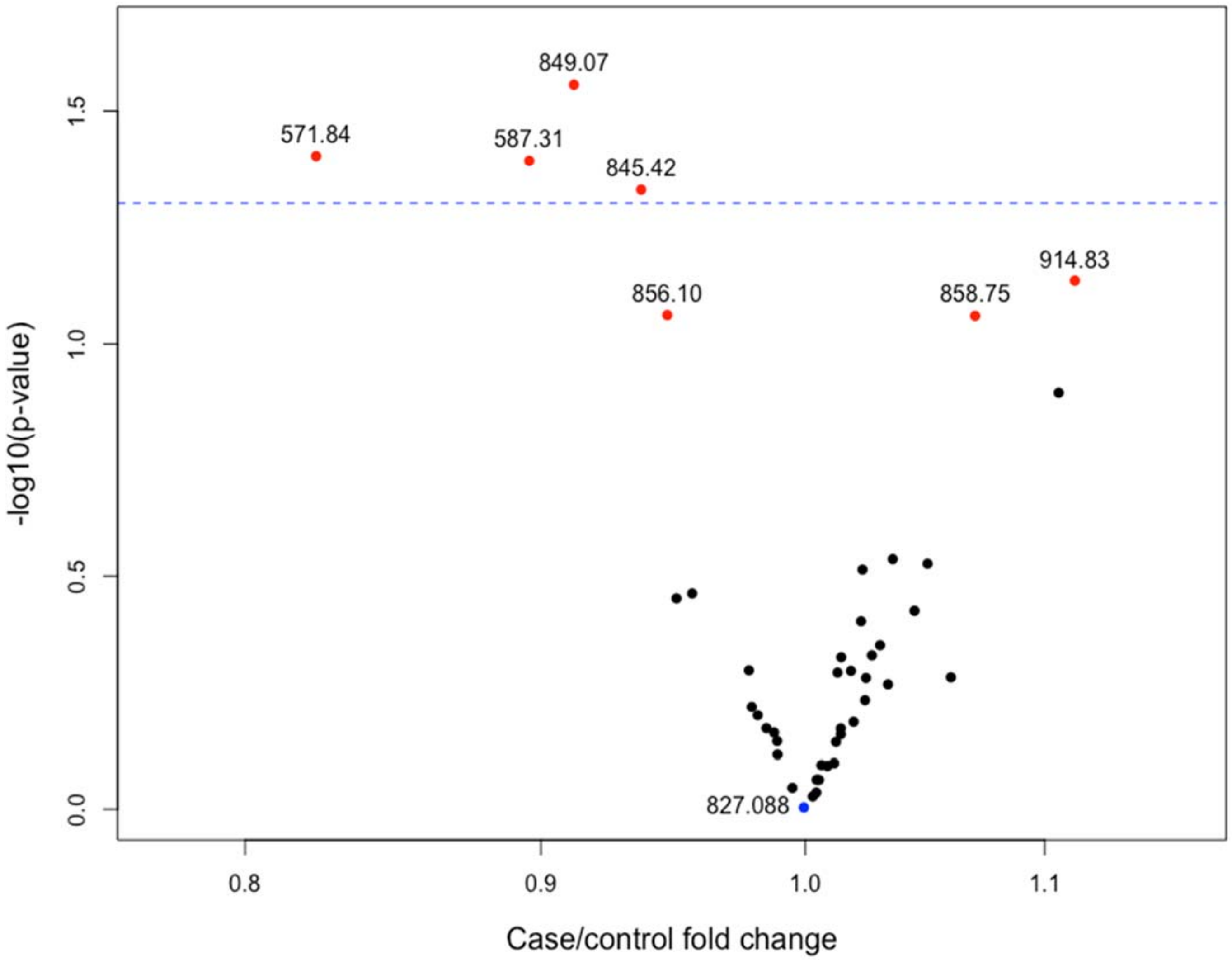
849.07
571.84
587.31
845.42
914.83
856.10
858.75
827.088
1.5
1.0
0.5
0.0
-log10(p-value)
0.8
0.9
1.0
1.1
Case/control fold change

B

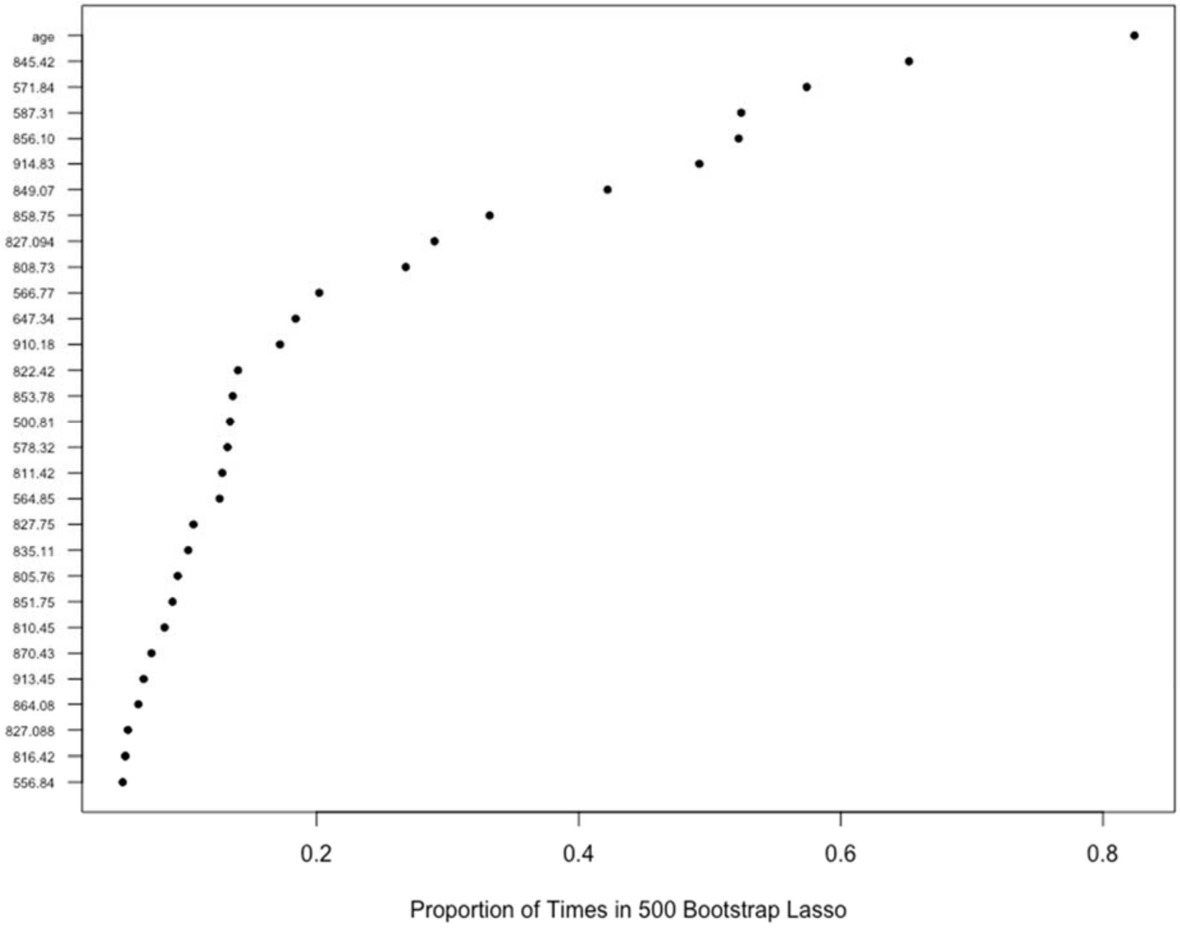

age
845.42
571.84
587.31
856.10
914.83
849.07
858.75
827.094
808.73
566.77
647.34
910.18
822.42
853.78
500.81
578.32
811.42
564.85
827.75
835.11
805.76
851.75
810.45
870.43
913.45
864.08
827.088
816.42
556.84
0.2
0.4
0.6
0.8
Proportion of Times in 500 Bootstrap Lasso

C

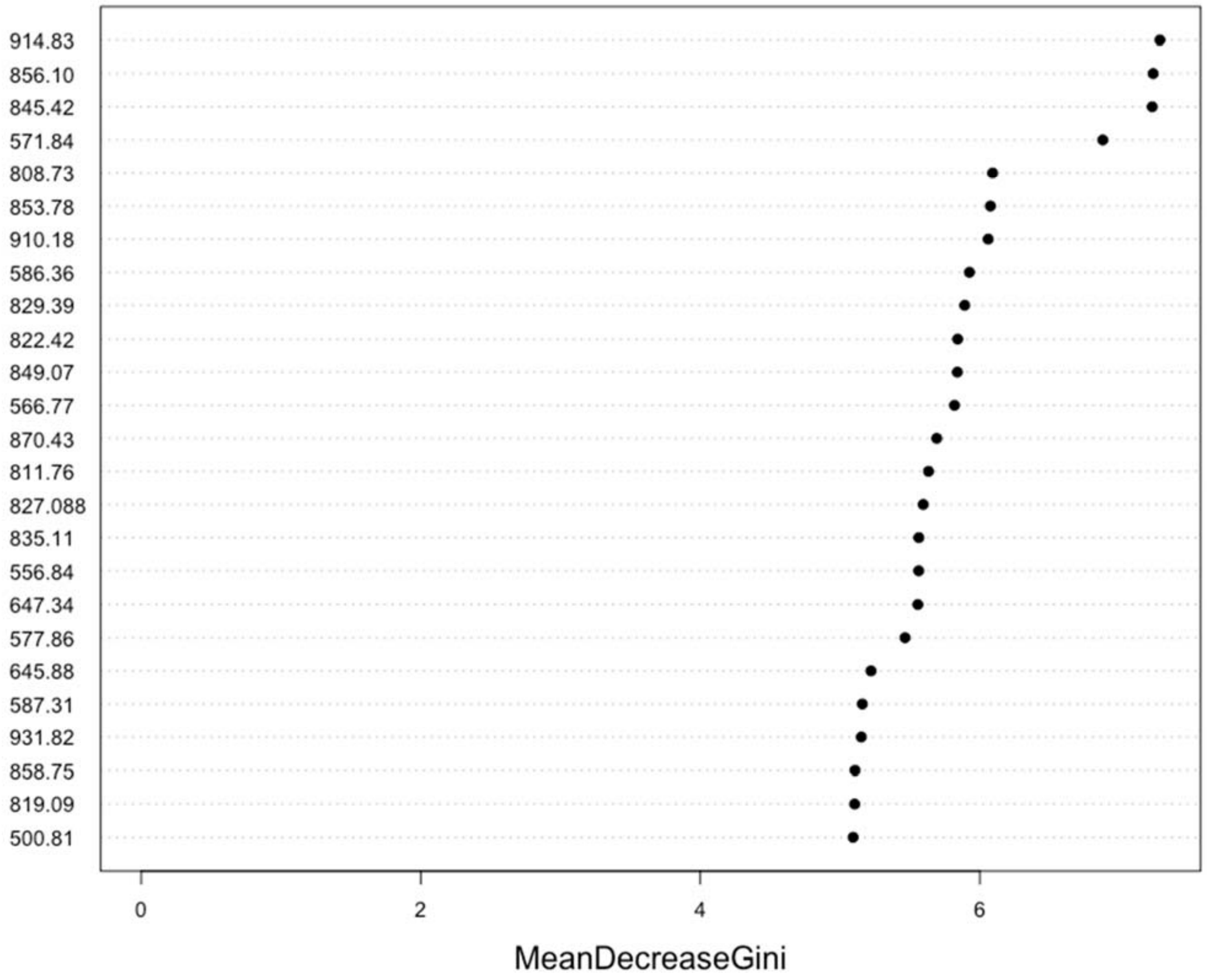

Supplementary Figure S2. Ensemble of regression and classification methods for selection of adduct features associated with case/control status for LUAD cases. A) Volcano plot of nominal *p*-values for the case/control status regression coefficient in a multivariate linear regression of each adduct's log abundance on case/control status and other covariates, with selected adducts shown in red (dashed line indicates *p*=0.05); B) proportion of times that a given adduct was selected by a regularized logistic regression (LASSO) of LUAD case/control status; C) ranked variable importance measures from a Random Forests classification of case/control status.

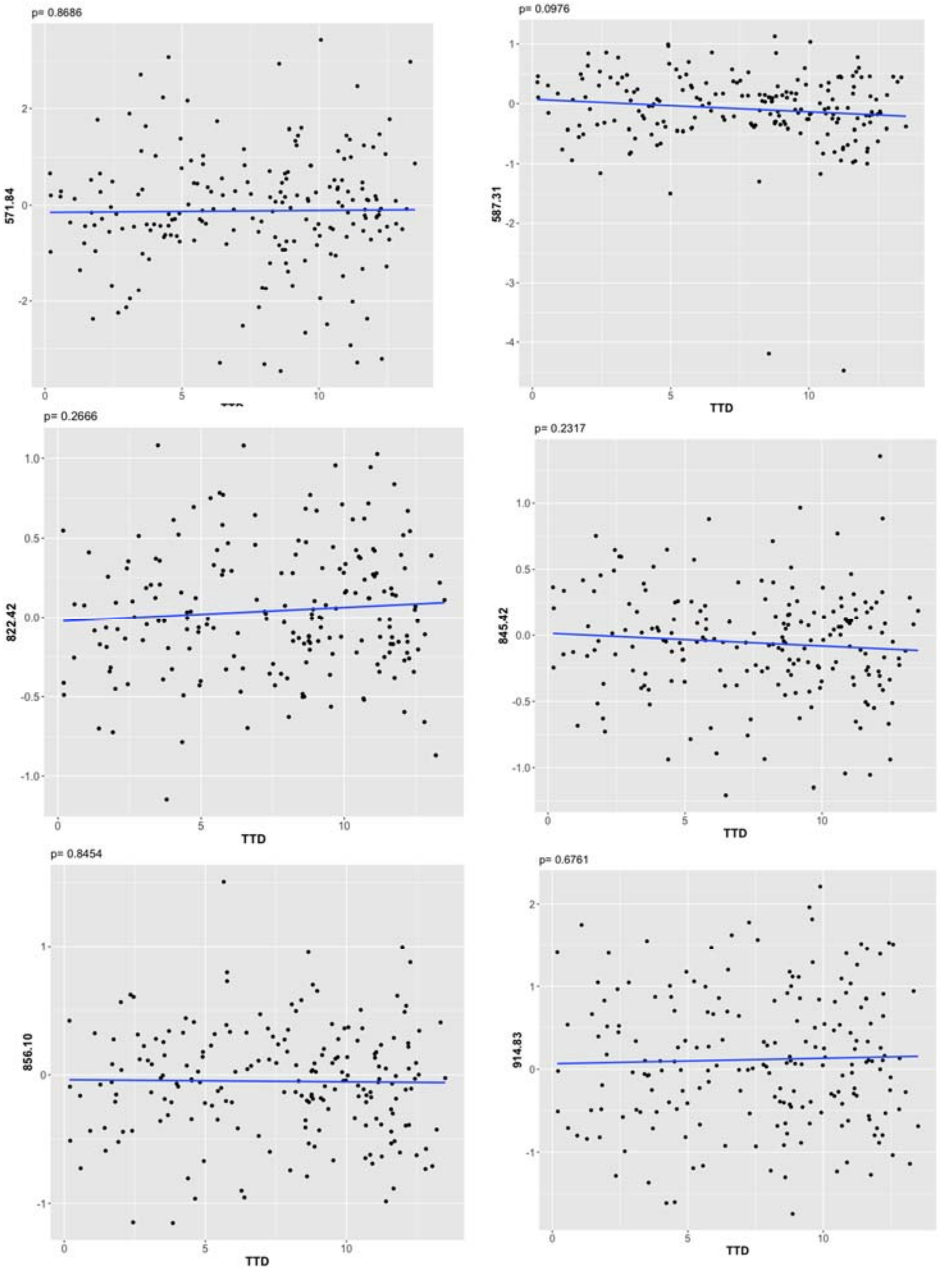

Supplementary Figure S3. Plots of ln(case/matched-control fold change) versus time to diagnosis (ttd) in years from recruitment for adduct features selected for association with histologically confirmed (HC) lung cancers. *P*-values are shown for slopes of the relationships.

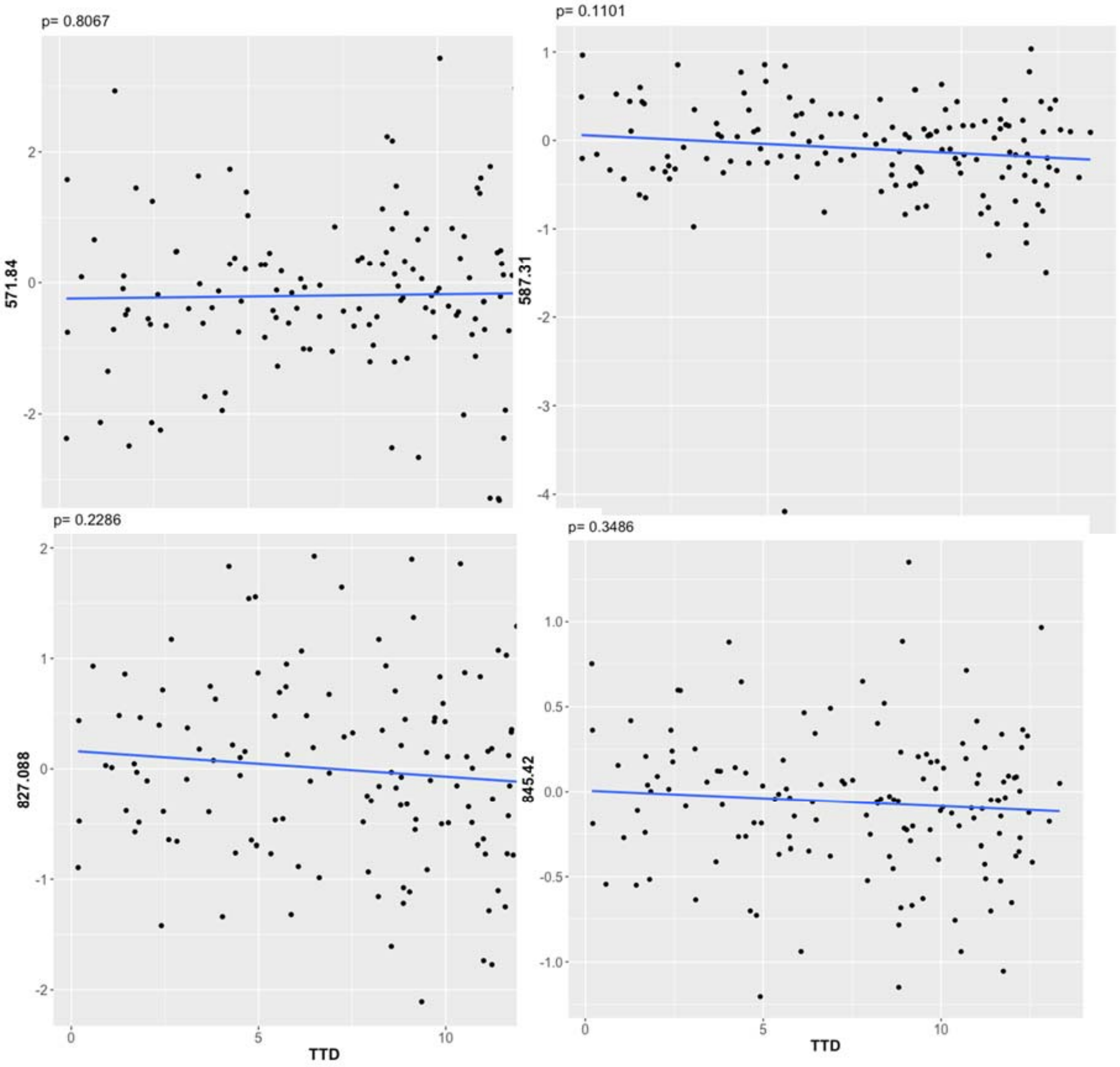
p= 0.8067
571.84
p= 0.1101
587.31
p= 0.2286
827.088
p= 0.3486
845.42
TTD
TTD

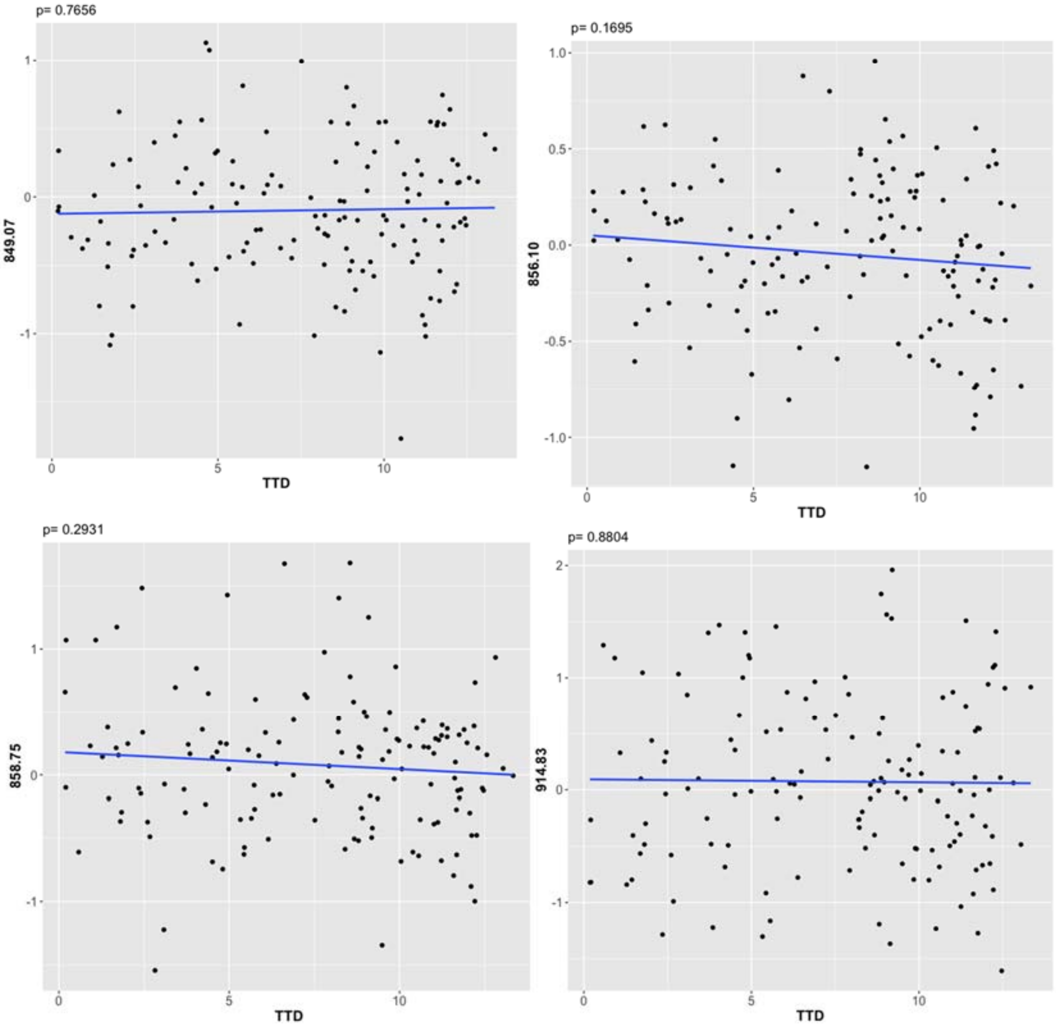

Supplementary Figure S4. Plots of ln(case/matched-control fold change) versus time to diagnosis (ttd) in years from recruitment for adduct features selected for lung adenocarcinoma (LUAD) cases/controls. *P*-values are shown for slopes of the relationships.
